# Radiation of the polymorphic Little Devil poison frog (*Oophaga sylvatica*) in Ecuador

**DOI:** 10.1002/ece3.3503

**Published:** 2017-10-18

**Authors:** Alexandre B. Roland, Juan C. Santos, Bella C. Carriker, Stephanie N. Caty, Elicio E. Tapia, Luis A. Coloma, Lauren A. O'Connell

**Affiliations:** ^1^ FAS Center for Systems Biology Harvard University Cambridge MA USA; ^2^ Department of Biological Sciences St. John's University Queens NY USA; ^3^ Lakeside High School Seattle WA USA; ^4^ Centro Jambatu de Investigación y Conservación de Anfibios Fundación Otonga Quito Ecuador

**Keywords:** amphibian, aposematism, ddRAD, Dendrobatidae, Ecuador, gene flow, *Oophaga sylvatica*, phenotypic variation, population genomics

## Abstract

Some South American poison frogs (Dendrobatidae) are chemically defended and use bright aposematic colors to warn potential predators of their unpalatability. Aposematic signals are often frequency‐dependent where individuals deviating from a local model are at a higher risk of predation. However, extreme diversity in the aposematic signal has been documented in poison frogs, especially in *Oophaga*. Here, we explore the phylogeographic pattern among color‐divergent populations of the Little Devil poison frog *Oophaga sylvatica* by analyzing population structure and genetic differentiation to evaluate which processes could account for color diversity within and among populations. With a combination of PCR amplicons (three mitochondrial and three nuclear markers) and genome‐wide markers from a double‐digested RAD (ddRAD) approach, we characterized the phylogenetic and genetic structure of 199 individuals from 13 populations (12 monomorphic and 1 polymorphic) across the *O. sylvatica* distribution. Individuals segregated into two main lineages by their northern or southern latitudinal distribution. A high level of genetic and phenotypic polymorphism within the northern lineage suggests ongoing gene flow. In contrast, low levels of genetic differentiation were detected among the southern lineage populations and support recent range expansions from populations in the northern lineage. We propose that a combination of climatic gradients and structured landscapes might be promoting gene flow and phylogenetic diversification. Alternatively, we cannot rule out that the observed phenotypic and genomic variations are the result of genetic drift on near or neutral alleles in a small number of genes.

## 1 INTRODUCTION

Aposematism is an adaptation that has evolved in many animals as a defense against predators (Caro, 2017). This strategy combines warning signals (e.g., vivid coloration) with diverse deterrents such as toxins, venoms, and other noxious substances. Most groups of animals include at least one example of aposematic lineage. Some examples of the most studied are *Heliconius* (Jiggins & McMillan, 1997) and Monarch butterflies (Reichstein, von Euw, Parsons, & Rothschild, 1968), nudibranch marine gastropods (e.g., *Polycera* Tullrot & Sundberg, 1991), *Plethodon* salamanders (Hensel & Brodie, 1976) and dendrobatid poison frogs (Myers & Daly, 1983; Santos, Coloma, & Cannatella, 2003; Saporito, Zuercher, Roberts, Gerow, & Donnelly, 2007), *Micrurus* coral snakes (Brodie, 1993), and *Pitohui* (Dumbacher, Beehler, Spande, Garraffo, & Daly, 1992) and *Ifrita* birds (Dumbacher, Spande, & Daly, 2000). The aposematism strategy is dependent on the predictability of the warning signal for effective recognition as well as learning and avoidance by predators (Benson, 1971; Chouteau, Arias, & Joron, 2016; Kapan, 2001; Ruxton, Sherratt, & Speed, 2004), with the expectation that common forms (i.e., similar looking) have a frequency‐dependent advantage on rarer forms in aposematic prey (Endler & Greenwood, 1988; Greenwood, Cotton, & Wilson, 1989). Many aposematic species present high levels of coloration and patterning polymorphisms (Mallet & Joron, 1999; Przeczek, Mueller, & Vamosi, 2008; Rojas, 2016), which in the context of mimicry, for example, *Heliconius* butterflies, might be under the control of a supergene (Joron et al., 2011). Nonetheless, the processes that produce and maintain such polymorphism in vertebrates remain a fundamental question in the evolutionary ecology of aposematism. To address this gap in our knowledge, studies on the population structure and biogeography of highly polymorphic and aposematic species are needed.

Neotropical poison frogs are endemic to Central and South America and evolved sequestration of chemical defenses coupled with warning coloration at least four times within the Dendrobatidae clade (Santos et al., 2014). Some poison frog genera/subgenera (a taxonomic and nomenclatural agreement is still pending, see Brown et al., 2011; Santos et al., 2009) display a wide range of inter‐ and intraspecific color and pattern variability, including *Dendrobates* sensu lato and all its proposed subclades (Noonan & Wray, 2006), such as *Adelphobates* (Hoogmoed & Avila‐Pires, 2012), *Excidobates*,* Andinobates*,* Ranitomeya* (Symula, Schulte, & Summers, 2001; Twomey, Vestergaard, & Summers, 2014; Twomey, Vestergaard, Venegas, & Summers, 2016; Twomey et al., 2013), and *Oophaga* (Brusa, Bellati, Meuche, Mundy, & Pröhl, 2013; Medina, Wang, Salazar, & Amézquita, 2013; Posso‐Terranova & Andrés, 2016a; Wang & Shaffer, 2008). Some species like *Ranitomeya imitator* have evolved variation in coloration and patterning resulting in Müllerian mimicry rings (Symula et al., 2001; Twomey et al., 2014). Among dendrobatids, the best‐known example of polymorphism is the strawberry poison frog, *Oophaga pumilio* (Schmidt, 1857), which is extremely variable in coloration within a small geographical area, that is, the archipelago of Bocas del Toro in Panamá (Daly & Myers, 1967; Summers, Cronin, & Kennedy, 2003). This observation of extreme polymorphism is evident when compared with the less variable coloration in most of its mainland populations distributed from eastern Nicaragua to western Panamá (Savage, 1968). Many factors might account for the origin and maintenance of color polymorphism in *O. pumilio*, including selective pressure from multiple predators, mate choice based on visual cues, and genetic drift variability among island populations of the archipelago (Gehara, Summers, & Brown, 2013; Tazzyman & Iwasa, 2010). However, how some *Oophaga* species maintain extreme coloration and patterning diversity without clear biogeographic barriers is still a mystery.

Many *Oophaga* species present a high level of phenotypic polymorphism, and molecular phylogenies show unclear species delimitation and suggest patterns of gene flow and hybridization. Currently, the *Oophaga* genus is composed of nine species, which have extraordinary morphological and chemical diversity (Daly, 1995; Daly, Brown, Mensah‐Dwumah, & Myers, 1978; Daly & Myers, 1967; Saporito, Donnelly, et al., 2007). Research conducted in *O. pumilio*, including a number of studies in population genetics, phylogeography, behavior, diet specialization, and chemical defenses (Dreher, Cummings, & Pröhl, 2015; Gehara et al., 2013; Richards‐Zawacki, Wang, & Summers, 2012; Saporito, Donnelly, et al., 2007), suggests that this species might include at least two distinctive mitochondrial lineages, each of which contain one or more congenerics: *O. speciosa*,* O. arborea*, or *O. vicentei* (Hagemann & Pröhl, 2007; Hauswaldt, Ludewig, Vences, & Pröhl, 2011; Wang & Shaffer, 2008). Moreover, the phylogeographic patterns observed in *O. pumilio* suggest a series of dispersals and isolations leading to allopatric divergence and then subsequent admixture and introgression among *Oophaga* species (Hagemann & Pröhl, 2007; Hauswaldt et al., 2011; Wang & Shaffer, 2008). A similar example of divergent evolution has been documented in *O. granulifera* along the Pacific coast of Costa Rica (Brusa et al., 2013). In this species, most of its populations also have variable levels of genetic admixture that might account for their phenotypic diversity.

Species boundaries are sometimes difficult to delineate, and extreme polymorphism within a species can reveal early processes of speciation events. This is especially evident in species with extreme polymorphism, but limited genetic characterization. For instance, *Oophaga* species restricted to South America have only been studied recently in terms of their population structure and natural history (Posso‐Terranova & Andrés, 2016a). Recent observations among the *Oophaga* distributed in Colombia (i.e., *O. histrionica* and *O. lehmanni*) suggest a complex pattern of diversification correlated with a structured landscape and strong shifts in climatic niches (Posso‐Terranova & Andrés, 2016b). These Colombian *Oophaga* were previously recognized as three nominal taxa, that is, *O. histrionica*,* O. occultator*, and *O. lehmanni* (Myers & Daly, 1976), but the former has long been suspected to be a species complex (Lötters, Glaw, Köhler, & Castro, 1999). This intuition was supported with genetic information, and *O. histrionica* has recently been proposed to include three new species (Posso‐Terranova & Andrés, 2016a).

Previous research in *O. histrionica* and *O. lehmanni* supports that hybridization among these species is an important process promoting color polymorphism (Medina et al., 2013). However, whether similar admixture or hybridization mechanisms also promote color polymorphisms within other highly polymorphic *Oophaga* is unknown, as genetic studies are absent. The Little Devil poison frog, *O. sylvatica* (Funkhouser, 1956), presents one of the most spectacular diversification patterns throughout its range. In addition, the presence of a highly polymorphic population restricted to a small geographic area represents a unique opportunity to further evaluate the role of selection pressures acting on the diversification of aposematic signals. We explored this complex population structure in *O. sylvatica* using a set of PCR amplicons (including mitochondrial and nuclear markers) and a collection of genome‐wide single‐nucleotide polymorphisms (SNPs) obtained from double‐digested RAD sequencing from 13 geographically distinct populations, among which 12 are monomorphic and one is polymorphic. The aim of our research was to explore the phylogeographical pattern of *O. sylvatica* along its distribution range in Ecuador by (1) estimating population structure and genetic differentiation and (2) evaluating which processes could account for color diversity within and among populations of *O. sylvatica*.

## 2 MATERIALS AND METHODS

### 2.1 Sample collection

**Figure 1:**
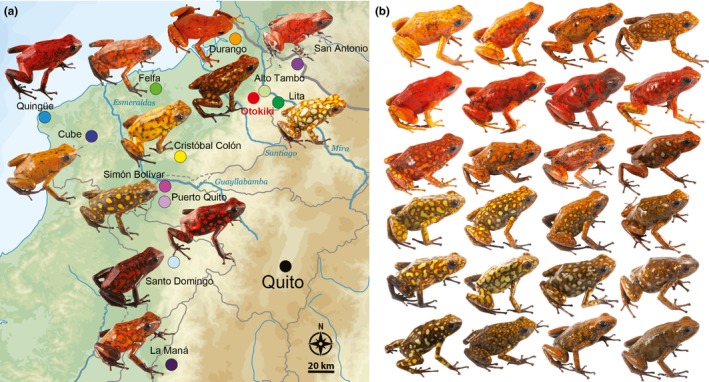
(a) *Oophaga sylvatica* distribution in Ecuador and morphological diversity. *Oophaga sylvatica* were found in lowland and foothill rain forest (0 to 1,020 m above sea level) in northwestern Ecuador. Most frogs were phenotypically variable among geographical localities (populations), while relatively monomorphic within populations (Fig. S1). Color diversity is particularly dramatic, ranging from yellow to red to brown and greenish, and can be combined with either markings or spots of different colors. (b) A striking example of diversity within the population of Otokiki, located in the center of the northern range, with phenotypes similar to the surrounding monomorphic populations as well as intermediate phenotypes

We sampled 13 populations of *O. sylvatica* (*N* = 199 individuals, detailed in Table 1) in July 2013 and July 2014 throughout its Ecuadorian distribution (Figure 1). Frogs were collected during the day with the aid of a plastic cup and stored individually in plastic bags with air and leaf litter for 3–8 hr. Individual frogs were photographed the day of capture in a transparent plastic box over a white background, then anesthetized with a topical application of 20% benzocaine to the ventral belly, and euthanized by cervical transection. Tissues were preserved in either RNAlater (Life Technologies, Carlsbad, CA, USA) or 100% ethanol. Muscle and skeletal tissue were deposited in the amphibian collection of Centro Jambatu de Investigación y Conservación de Anfibios in Quito, Ecuador (Appendix S2, Table S1). The Institutional Animal Care and Use Committee of Harvard University approved all procedures (Protocol 15–02–233). Collection and exportation of specimens were performed under permits (001–13 IC‐FAU‐DNB/MA, CITES 32 or 17 V/S) issued by the Ministerio de Ambiente de Ecuador. Samples from *O. histrionica* (two individuals from Colombia: Chocó: Quibdo, La Troje; voucher numbers TNHCFS4985 [longitude: −76.591, latitude: 5.728] and TNHCFS4987 [longitude: −76.591, latitude: 5.728]) and *O. pumilio* (six individuals from three different populations, El Dorado, Vulture Point, and Almirante acquired from the USA pet trade) were treated using the same protocol. In order to protect the vulnerable *O. sylvatica* populations that are highly targeted by illegal poaching, specific GPS coordinates of frog collection sites can be obtained from the corresponding author.

**Table 1:** Summary of species and within‐population diversity for the concatenated mitochondrial genes (12S‐tRNA^Val^, 16S, CO1)

Marker	Species/population	*N*	*S*	*H*	Hd	*K*	π
Mitochondrial (2,084 bp)	*Oophaga sylvatica*	199	83	61	0.94863	11.66327	0.0056
	Durango	14	8	6	0.85714	3.34066	0.00161
	Lita	6	8	4	0.86667	3.8	0.00183
	Alto Tambo	6	12	5	0.93333	6.06667	0.00292
	Otokiki	83	46	29	0.95386	8.36879	0.00402
	San Antonio	14	22	6	0.6044	5.82418	0.00281
	Felfa	9	16	4	0.69444	3.83333	0.00185
	Quingüe	8	1	2	0.42857	0.42857	0.00021
	Cube	7	2	3	0.66667	0.7619	0.00037
	Cristóbal Colón	10	6	4	0.77778	2.15556	0.00104
	Simón Bolívar	13	9	5	0.75641	3	0.00137
	Puerto Quito	8	4	3	0.67857	1.85714	0.0009
	Santo Domingo	8	2	3	0.60714	0.67857	0.00033
	La Maná	13	0	1	0	0	0
	*O. histrionica*	2	0	1	0	0	0
	*O. pumilio*	6	30	4	0.86667	15.8	0.00697
	Overall	207	163	66	0.95239	13.31143	0.00753

*N*, number of individuals sequenced; *S*, number of segregating sites; *H*, number of haplotypes; Hd, haplotype diversity; *K*, sequence diversity; π, nucleotide diversity.

### 2.2 DNA extraction and amplification

Liver or skin tissue stored in RNAlater was homogenized using Trizol (Life Technologies) in tubes with 1.5‐mm TriplePure Zirconium Beads (Bioexpress, Kaysville, UT, USA). DNA was purified by a standard phenol/chloroform procedure followed by ethanol precipitation according to the Trizol manufacturer's instructions.

Three mitochondrial (cytochrome oxidase subunit 1 [CO1 or *cox1*], 16S ribosomal DNA [16S], and 12S ribosomal DNA [12S] flanked with tRNA^Val^) and three nuclear (recombination‐activating gene 1 [RAG‐1], tyrosinase [TYR], and sodium‐calcium exchanger 1 [NCX]) gene regions were PCR‐amplified using the following sets of primers for each gene: CO1 (CO1a‐H: 5′‐AGTATAAGCGTCTGGGTAGTC and CO1f‐L: 5′‐CCTGCAGGAGGAGGAGAYCC) (Palumbi et al., 2002), 16S (16sar‐L: 5′‐CGCCTGTTTATCAAAAAC and 16sbr‐H: 5′‐CCGGTCTGAACTCAGATCACGT) (Palumbi et al., 2002), 12S‐tRNA^Val^ (MVZ59‐L: 5′‐ATAGCACTGAAAAYGCTDAGATG and tRNAval‐H: 5′‐GGTGTAAGCGARAGGCTTTKGTTAAG) (Santos & Cannatella, 2011), RAG‐1 (Rag1_Oop‐F1: 5′‐CCATGAAATCCAGCGAGCTC and Rag1_Oop‐R1: 5′‐CACGTTCAATGATCTCTGGGAC) (Hauswaldt et al., 2011), TYR (TYR_Oosyl_F: 5′‐AACTCATCATTGGGTTCACAATT and TYR_Oosyl_R: 5′‐GAAGTTCTCATCACCCGTAAGC), and NCX (NCX_Oosyl_F: 5′‐ACTATCAAGAAACCAAATGGTGAAA and NCX_Oosyl_R: 5′‐TGTGGCTGTTGTAGGTGACC). NCX and TYR primers were designed from publicly available *O. sylvatica* sequences (GenBank accession numbers HQ290747 and HQ290927).

DNA was amplified in a 30 μl PCR containing 10 ng of genomic DNA, 200 nmol/L of each primer, and 1× Accustart II PCR SuperMix (Quanta Biosciences, Gaithersburg, MD, USA). The thermocycling profiles comprised an initial denaturation (3 min at 95°C), followed by 40 cycles of denaturation (30 s at 95°C), annealing (30 s) with specific temperature for each primer set (see below), elongation (72°C) with a duration specific to each primer set (see below), and a final elongation step (5 min at 72°C). Specific parameters for annealing temperature (*T*_a_) and elongation time (*E*) for each primer set are as follows: 16S (*T*_a_ = 50°C, *E* = 45 s), CO1 (*T*_a_ = 54°C, *E* = 45 s), 12S (*T*_a_ = 46°C, *E* = 60 s), RAG1 (*T*_a_ = 62°C, *E* = 45 s), TYR (*T*_a_ = 55°C, *E* = 40 s), NCX (*T*_a_ = 55°C, *E* = 80 s). PCR products were analyzed on an agarose gel and purified using the E.Z.N.A Cycle‐Pure Kit following the manufacturer protocol (Omega bio‐tek, Norcross, GA, USA). Purified PCR products were Sanger sequenced by GENEWIZ (South Plainfield, NJ, USA).

### 2.3 ddRADseq library generation and sequencing

We constructed double‐digested restriction‐site‐associated DNA sequencing (ddRAD) libraries on a subset of 125 samples of *O. sylvatica* following the protocol in Peterson, Weber, Kay, Fisher, and Hoekstra (2012). Samples include three specimens randomly drawn within each sampling site from the monomorphic populations (with two sampling sites for Durango and San Antonio populations) and all the specimens from the polymorphic region in Otokiki (see Figure 1). DNA was extracted from skin tissues preserved at −20°C in RNAlater using the NucleoSpin DNA kit (Macherey‐Nagel, Bethlehem, PA, USA). Genomic DNA of each sample (1 μg) was digested using 1 μl of EcoRI‐HF (20,000 U/ml) and 1 μl of SphI‐HF (20,000 U/ml) (New England Biolabs, Ipswitch, MA, USA) following the manufacturer's protocol. Digested samples were then cleaned with Agencourt Ampure XP beads (Beckman Coulter, Danvers, MA, USA). Purified digested DNA (100 ng) was ligated to double‐stranded adapters (biotin‐labeled on P2 adapter) with a unique inline barcode using T4 DNA ligase (New England Biolabs) and purified with Agencourt Ampure XP beads. Barcoded samples were pooled and size‐selected between 250 and 350 bp (326–426 bp accounting for the 76 bp adapter) using a Pippin Prep 2% agarose gel cassette (Sage Science, Beverly, MA, USA). Sized‐selected fragments were purified with Dynabeads MyOne Streptavidin C1 (Life Technologies). Samples were divided into three independent libraries and amplified using Phusion High‐Fidelity DNA polymerase (New England Biolabs) for 12 cycles. Libraries were then pooled and cleaned with Agencourt Ampure XP beads. Paired‐end sequencing (125 bp) was conducted on an Illumina HiSeq 2500 at the FAS Bauer Core Facility at Harvard University.

### 2.4 Analysis of mitochondrial and nuclear markers

Raw sequence chromatograms for each gene set were edited and trimmed using GENEIOUS 8.0.5 (BioMatters Ltd., Auckland, New Zealand) and then aligned using CLUSTALW. Alleles of nuclear genes containing heterozygous sites were inferred using a coalescent‐based Bayesian method developed in PHASE 2.1 (Stephens, Smith, & Donnelly, 2001) as implemented in DNASP 5.10.01 (Librado & Rozas, 2009). Three independent runs of 10,000 iterations and burn‐in of 10,000 generations were conducted to check for consistency across runs.

The best‐fitting substitution models for each mitochondrial data set (12S‐tRNA^Val^, 16S, CO1) and for the concatenated matrix of 2,084 bp were determined with JMODELTEST 2.1 (Darriba, Taboada, Doallo, & Posada, 2012). The Hasegawa–Kishino–Yano (HKY) model with a proportion of invariable sites (+I) was selected based on Bayesian information criterion (BIC) and decision theory, with the exception of the 16S data set supported by Akaike information criterion only.

Mitochondrial genes were then considered as a single unit for each individual of the different populations of *O. sylvatica* (*N* = 199), *O. histrionica* (*N* = 2), and *O. pumilio* (*N* = 6). Diversity indices were calculated using DNASP, and a mitochondrial haplotype network was inferred under Tajima and Nei model using ARLEQUIN 3.5 (Excoffier & Lischer, 2010) and drawn using GEPHI (Bastian, Heymann, & Jacomy, 2009), under the ForceAtlas2 algorithm in default settings.

We calculated population differentiation using conventional F‐statistics from haplotype frequencies (*F*_ST_) and genetic distances based on pairwise difference (Φ_ST_) using ARLEQUIN. *p*‐Values for *F*_ST_ and Φ_ST_ were estimated after 10,000 permutations, and significance threshold level was fixed at *p* = .05.

### 2.5 ddRAD sequence analysis

Raw fastq reads were demultiplexed, quality filtered for reads with Phred quality score <20, and trimmed to 120 bp using the *process*_*radtags.pl* command from the STACKS 1.35 pipeline (Catchen, Hohenlohe, Bassham, Amores, & Cresko, 2013). ddRAD loci were constructed de novo with the STACKS *denovo_map* function (parameters: *m* = 5, *M* = 3, *n* = 3) and then corrected for misassembled loci from sequencing errors using the corrections module (*rxstacks*,* cstacks*, and *sstacks*), which applies population‐based corrections. In order to minimize the effect of allele dropout that generally leads to over‐estimation of genetic variation (Gautier et al., 2013), we selected loci present in at least 75% of the individuals of each of the 13 sampled populations and generated population statistics and output files using STACKS *population* pipeline (parameters: *r* = 0.75, *p* = 13, *m* = 5). The data set used for the subsequent analysis was filtered to retain only one random SNP site per RAD locus to minimize within‐locus linkage and is composed of 3,785 SNPs.

Genetic structure and individual assignments were investigated using Bayesian clustering methods implemented in the program STRUCTURE 2.3.4. The software assumes a model with *K* populations (where *K* is initially unknown), and individuals are then assigned probabilistically to one or more populations (Pritchard, Stephens, & Donnelly, 2000). We ran the admixture model with correlated allele frequencies (Falush, Stephens, & Pritchard, 2007) for 100,000 burn‐in and 1,000,000 sampling generations for *K* ranging from one to the number of sampled population plus three (*K* = 1–16) with 10 iterations for each value of *K*. We determined the number of clusters (*K*) that best described the data using the delta *K* method (Evanno, Regnaut, & Goudet, 2005) as implemented in STRUCTURE HARVESTER (Earl & vonHoldt, 2012) and analyzed the results using CLUMPAK (Kopelman, Mayzel, Jakobsson, Rosenberg, & Mayrose, 2015).

As the previous approach relies on a particular population genetic model where populations meet Hardy–Weinberg and linkage equilibriums, we used an assumption‐free multivariate method implemented in the R package “*adegenet”* (Jombart & Ahmed, 2011). The discriminant analysis of principal components (DAPC), which is also suitable for analyzing large numbers of SNPs (Jombart, Devillard, & Balloux, 2010), provides an assignment of individuals to groups and a visual assessment of between‐population differentiation. To avoid over‐fitting of the discriminant functions, we performed a stratified cross‐validation of DAPC using the function *xvalDapc* from “*adegenet”* and retained 20 principal components, which gave the highest mean success and the lowest root‐mean‐squared error. Finally, we plotted the membership probability of each individual using the *compoplot* function with two discriminant analyses.

### 2.6 Spatial patterns of genetic variability

We investigated the general pattern of isolation by distance for each set of mitochondrial markers separately using Mantel test (Mantel, 1967) and Mantel correlogram from the R package “*vegan”* (Oksanen, Blanchet, Kindt, Legendre, & O'Hara, 2016), with geographic distance calculated using the “*geosphere”* package (Hijmans, Williams, & Vennes, 2014). Specifically, we used the function distVincentyEllipsoid “Vincenty” (ellipsoid) great circle distance to calculate the shortest distance between two points, for example, two geographic coordinates, according to the “Vincenty (ellipsoid)” method. This method is very accurate, yet it does not include Earth's topography. We are aware that altitude might influence the distance, but there is currently not a bioinformatics implementation for the Tropics. We then investigated the spatial pattern of genetic variability among populations using the spatial principal component analysis (sPCA) implemented in the “*adegenet”* (Jombart, Devillard, Dufour, & Pontier, 2008) for the ddRAD data set. This package implements a multivariate analysis without prior assumption of genetic models to reveal global and local structure patterns.

## 3 RESULTS

### 3.1 Mitochondrial and nuclear amplicon diversity and structure

**Figure 2:**
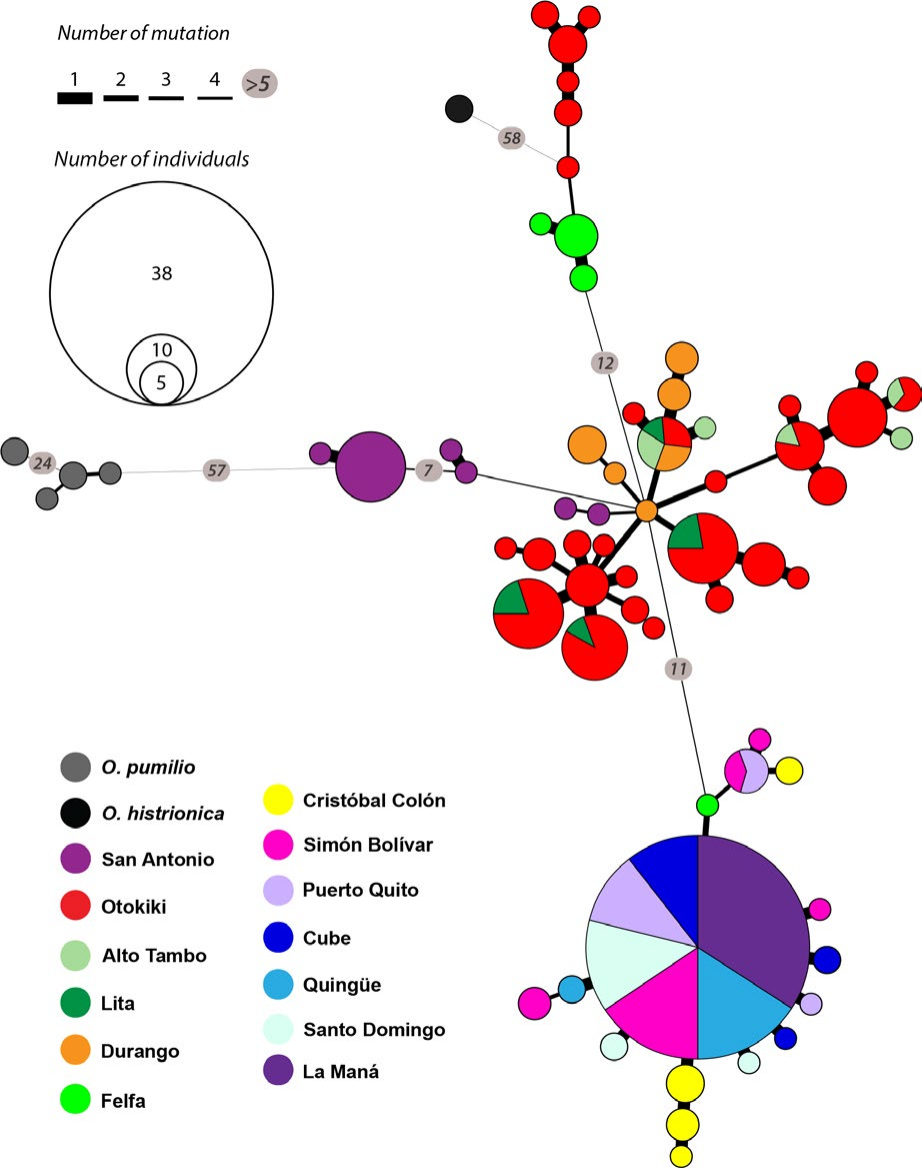
Haplotype network of 66 unique haplotypes of concatenated mitochondrial genes (12S‐tRNA^V^ ^al^, 16S, CO1) of *Oophaga sylvatica*,* O. histrionica*, and *O. pumilio* (2,084 bp). Circles indicate haplotypes, with the area being proportional to the number of individuals sharing that haplotype. Colors refer to the geographic origin of the population, and the pie charts represent the percentage of each population sharing the same haplotype. Line thickness between haplotypes is proportional to the inferred mutational steps (or inferred intermediate haplotypes). Inferred numbers of mutational steps are shown inside circles along the line when greater than four steps

Among the 13 populations of *O. sylvatica* sampled, genetic diversity is relatively high, with 83 segregating sites, 61 haplotypes, and a mean haplotype diversity (Hd) of 0.948 (Table 1 and Figure 2). Both sister species *O. histrionica* and *O. pumilio* are well separated from *O. sylvatica* (Figure 2). These branches constitute two remote clusters, one composed of haplotypes from Otokiki and Felfa populations (for their geographic location, see Figure 1) that link to *O. histrionica*, and one composed of haplotypes from the San Antonio population that link to *O. pumilio*. Both branches link to the same node, a haplotype of one individual from Durango. Six short branches (two to four inferred mutations) radiate from this central node: Two are composed of private haplotypes from the Durango and San Antonio populations, and the other four have mixed origins belonging to the northern populations of Otokiki, Durango, Lita, and Alto Tambo. These populations show the highest sequence (*K*) and nucleotide (π) diversity, as well as the highest haplotype diversity (Table 1). One of the mixed clusters includes haplotypes from Durango, Lita, Alto Tambo, and Otokiki populations; another includes haplotypes from Alto Tambo and Otokiki, and two different clusters are composed of haplotypes from the Otokiki and Lita populations. All of these clusters have interhaplotype distances ranging from one to four inferred mutational steps. With 29 haplotypes, Otokiki is the most genetically diverse population (Table 1 and Figure 2), in addition to exhibiting the most variable color patterning (i.e., polymorphic) (Figure 1b). The color variations seem to be a combination of Alto Tambo, Lita, and Durango (Figure 1a), the geographically closest populations that are also comparatively monomorphic (Appendix S1, Fig. S1). Six haplotypes are shared between Otokiki and at least one of these closer monomorphic populations, and one haplotype is shared by all populations.

We found that haplotype clusters follow a geographic pattern roughly separating “northern” and “southern” populations. These two large groups of haplotypes are linked through an individual from Felfa clustering with the southern populations. Southern populations have less genetic diversity than northern populations, where 67 individuals collapsed in 15 haplotypes (Figure 2). One branch is composed of a small group of three haplotypes from the Puerto Quito, Simón Bolívar, and Cristóbal Colón populations. The second branch leads to one haplotype including 38 individuals from 6 populations: Quingüe, Cube, Simón Bolívar, Puerto Quito, Santo Domingo, and La Maná. Radiating from this haplotype are 11 unique haplotypes (one to four individuals each) with mostly 1 inferred mutational step. Among them, the frogs from the Cristóbal Colón population do not share any haplotype with the rest of the southern populations.

Interestingly, despite conducting an evaluation of genetic structure, nuclear amplicons did not show any phylogeographic patterns through haplotype and STRUCTURE analyses. Thus, those results are presented as supplementary documents in Appendix S1, Figs S2 and S3, respectively, including the calculated diversity indices (see Appendix S2, Table S2). The contrasting information, when compared with the mitochondrial and ddRAD data sets, is nevertheless not further discussed as it does not provide meaningful insight.

### 3.2 Population differentiation using mtDNA

**Figure 3:**
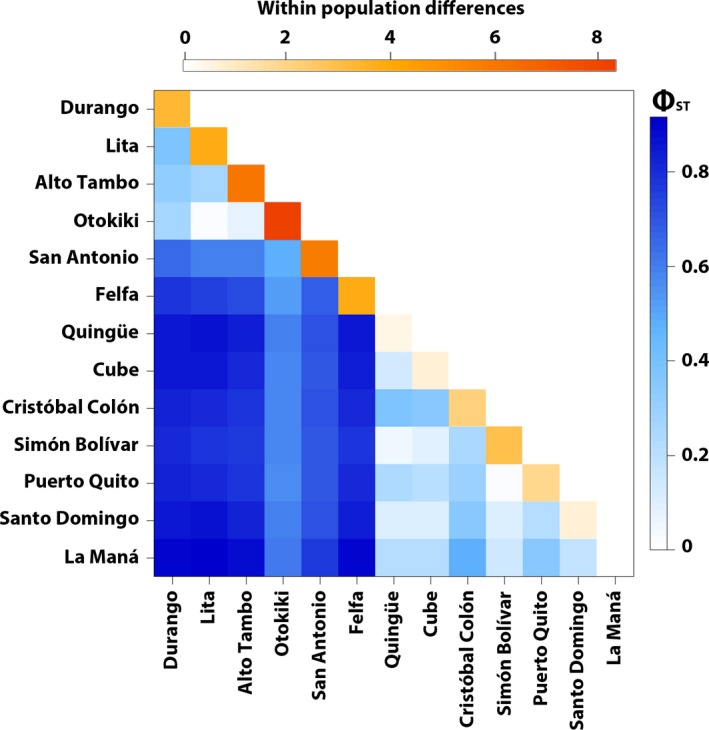
Heatmap representation of between and within‐population differentiation in *Oophaga sylvatica* for concatenated mitochondrial genes (12S‐tRNA^V^ ^al^, 16S, CO1). Below the diagonal are the pairwise ϕ_ST_ values between populations ranging from low (white) to high (blues). The diagonal is within‐population pairwise difference values ranging from low (white) to high (orange)

Levels of population differentiation are relatively high in *O. sylvatica*, with a mean *F*_ST_ of 0.220 (ranging from −0.011 to 0.705) and a mean Φ_ST_ of 0.609 (ranging from 0.016 to 0.915), suggesting that northern populations are well differentiated from southern populations (Figure 3). In the northern populations, we observe very low values of both *F*_ST_ and Φ_ST_ between the populations of Durango, Lita, Alto Tambo, and Otokiki. These values are statistically nonsignificant (*p*‐value* *> .05 after 10,000 permutations) for Otokiki versus Lita and Alto Tambo (both *F*_ST_ and Φ_ST_), and for Durango versus Alto Tambo and Lita versus Alto Tambo (*F*_ST_ only) (Appendix S2, Table S3). In the southern populations, frogs from Quingüe, Cube, Simón Bolívar, Puerto Quito, Santo Domingo, and La Maná are genetically similar in haplotype clustering (Figure 3) and this observation is confirmed by low *F*_ST_ and Φ_ST_ and nonsignificant *p*‐values (Appendix S2, Table S3). Finally, based on both *F*_ST_ and Φ_ST_ values, populations from San Antonio, Felfa, and Cristóbal Colón appear to be different from every other northern and southern population.

### 3.3 Population structure based on ddRAD markers

**Figure 4:**
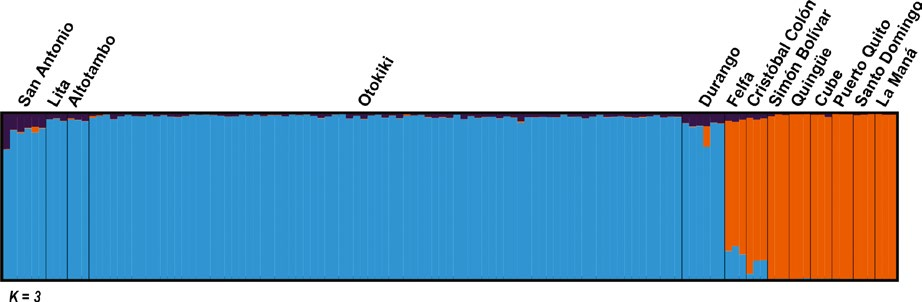
Structure inferred for 13 populations of *Oophaga sylvatica* from ddRAD data. Bar plots show Bayesian assignment probabilities for 125 individual frogs as inferred by STRUCTURE for *K* = 3 clusters, each color depicting one of the putative clusters

The optimal number of clusters inferred by Evanno's method for the 125 individuals of *O. sylvatica* in the ddRAD data set was *K* = 3 (see Figure 4 for colors assigned to clusters). We can observe a clear genetic structure with two main clusters. One genetic cluster (blue) represents mostly populations from the northernmost part of the range (San Antonio, Lita, Alto Tambo, Durango, and Otokiki). A second genetic cluster (orange) appears mostly in populations from the southern part of the range (Cristóbal Colón, Simón Bolívar, Quingüe, Cube, Puerto Quito, Santo Domingo, and La Maná), with the exception of Felfa that is distributed in the north. Finally, a third genetic cluster (purple) is present in small proportions in every population. In addition, two populations with intermediate range (Felfa and Cristóbal Colón) have admixed proportion of both main clusters (blue and orange).

### 3.4 Discriminant analysis of principal components on the ddRAD data

**Figure 5:**
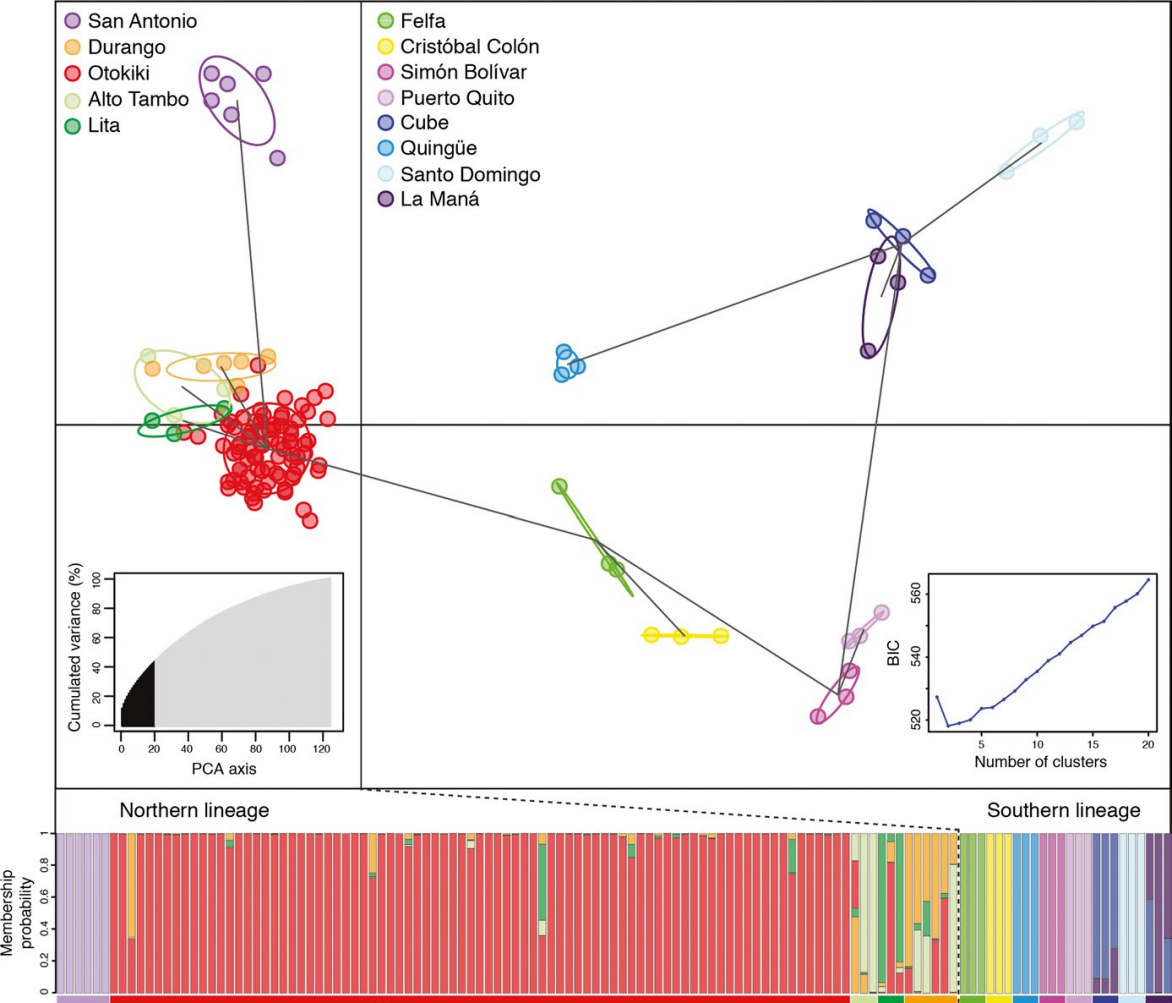
DAPC scatterplot for ddRAD data. The scatterplot shows the first two principal components of the DAPC of data generated with 3,785 SNPs. Individuals are represented by dots, and groups (i.e., geographic populations) are color‐coded according to Figure 1 and depicted by 95% inertia ellipses, which represent graphical summaries of clouds of points. Lines between groups represent the minimum spanning tree based on the squared distances and show the actual proximities between populations within the entire space. Right inset shows the inference of the number of clusters using the Bayesian information criterion (BIC). The chosen number of clusters corresponds to the inflexion point of the BIC curve (*K* = 2). Left inset shows the number of PCA eigenvalues retained in black and how much they accounted for variance. The bottom graph represents the membership probability of each individual to one or more populations. Geographic populations (groups) are represented with the same colors as in the DAPC plot

We conducted a DAPC analysis (Figure 5) and found an optimal number of clusters at *K* = 2 under the BIC. The two clusters follow the north/south split observed previously. In the north, individuals from the populations of Lita, Alto Tambo, and Durango overlap with individuals from Otokiki, but San Antonio is well separated from them. In the south, populations are separated from each other and individuals did not overlap with any other population. The membership probability of each individual shows overlapping individuals from Lita, Alto Tambo, Durango, and Otokiki, suggesting possible gene flow occurring among these populations (Figure 5). In addition, some individuals from the southern populations of Cube and La Maná have been assigned with mixed proportions to both populations.

### 3.5 Spatial pattern of genetic variability

**Figure 6:**
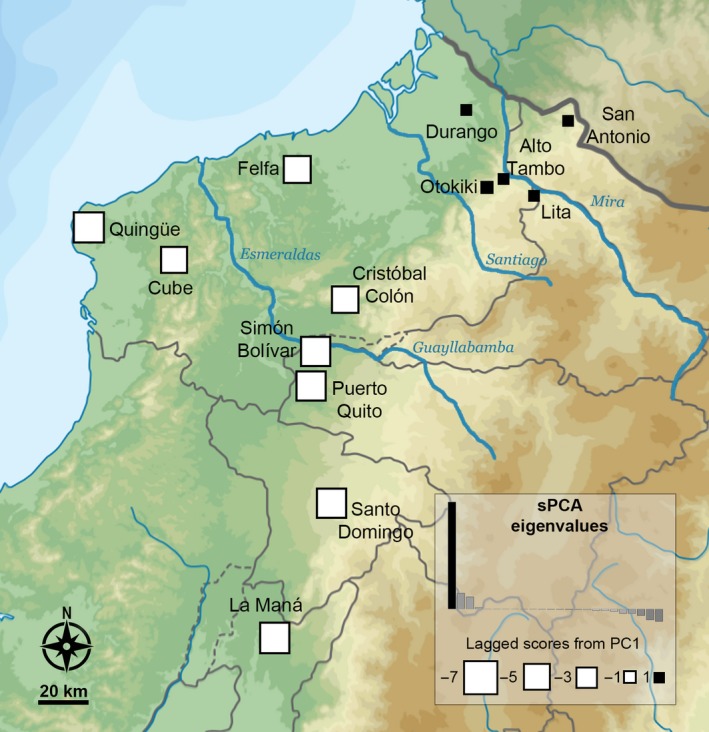
Analysis of the spatial pattern of genetic variability among 13 populations of *Oophaga sylvatica*. The map shows the results of the sPCA represented by the lagged scores (~principal component [PC] scores). The inset shows the sPCA eigenvalues, and only the PC associated with the first positive eigenvalue was retained (depicted by the black bar)

The Mantel tests conducted on each mitochondrial marker (Appendix S2, Table S4) suggest that the genetic pattern observed is due to isolation by distance, while mantel correlograms show that genetic distance is not always correlated with geographic distance (Appendix S1, Fig. S4). The sPCA analysis shows that the genetic pattern is better described by the first global score (one positive eigenvalue) and reveals a positive spatial autocorrelation between individuals within two clearly distinct patches (Figure 6). These patches correspond to the northern and southern genetic clusters identified previously and suggest that they do not result from isolation by distance, but likely due to landscape structure.

## 4 DISCUSSION

In this study, we investigated genetic structure among populations of *O. sylvatica* from northwestern Ecuador. Our results show that genetic structure of the populations is roughly split along their geographical distribution resulting in two main lineages: northern and southern genetic clusters separated at the Santiago River drainage (Figure 6). The existence of these two distinct lineages is supported by mitochondrial haplotype network (Figure 2), Bayesian assignment of individuals using Structure (Figure 4), DAPC analysis (Figure 5), and ddRADseq‐based phylogeny (Appendix S1, Fig. S5). Within each lineage, most populations share mitochondrial haplotypes (with the exception of San Antonio and Felfa in the north, and Cristóbal Colón in the south) and have low genetic differentiation (nonsignificant *F*_ST_ values).

The northern genetic cluster (San Antonio, Lita, Alto Tambo, Durango, and Otokiki) presents higher mitochondrial diversity with a large amount of unique haplotypes within populations. We observe that four of these populations (San Antonio, Lita, Alto Tambo, and Durango) have unique haplotypes, and three (Durango, Lita, and Alto Tambo) share haplotypes with the polymorphic population at Otokiki. These latter populations are weakly differentiated based on genetic data and are geographically close, with distances from Otokiki being, respectively, 4, 5, and 20 km to Alto Tambo, Lita, and Durango (Figure 6). Otokiki individuals present every color pattern found in the surrounding populations in addition to intermediate patterns (Figure 1). The DAPC plot highlights a genetic overlap between some individuals of Otokiki and the three populations from Lita, Alto Tambo, and Durango; some individuals were assigned with mixed proportions to these different groups (Figure 5). We acknowledge that the ddRAD data set by itself, which includes only three individuals of each monomorphic population, limits the strength of the conclusion we can draw. Nevertheless our results show a poor genetic differentiation associated with a mixture of phenotypes among populations with overlapping distributions. At least two scenarios could possibly explain the patterns that we observed. First, these features support the presence of a secondary contact zone occurring within Otokiki, which in turn may promote the dramatic color diversity of this population. Alternatively, Otokiki could be a large source population where surrounding populations represent recent expansions with less genetic and phenotypic diversity due to founder effects or other selection pressures. Although outside the scope of the current study, more sampling would allow a contrast of the allele frequency spectrum along a transect across the northern populations to help disentangle these alternative hypotheses.

The southern genetic cluster, composed of Felfa, Cristóbal Colón, Simón Bolívar, Quingüe, Cube, Puerto Quito, Santo Domingo, and La Maná, shows a large degree of color diversity between its populations but little mitochondrial diversity. Only a few haplotype variants radiate from a common haplotype shared by a majority of individuals across the different populations (Figure 2). Those populations are geographically distant from each other, ranging from 20 km between Simón Bolívar and Puerto Quito to as much as 190 km between Quingüe and La Maná, have low but significant *F*_ST_ values from ddRAD data (Appendix S2, Table S5), and are genetically well differentiated on the DAPC analysis. As amphibians usually have low dispersal abilities (Zeisset & Beebee, 2008) and Dendrobatidae, like most frogs, are not known to be migratory species, the low levels of differentiation observed with the mitochondrial markers are unlikely to have occurred as a result of gene flow. In addition, this low genetic variation combined with high phenotypic diversity among geographically distant population suggests that the southern cluster likely had a rapid radiation occurring within the last 2.1–2.5 Ma (Santos et al., 2009).

Repeated phylogeographical patterns of diversification among amphibians and reptiles across northwestern Ecuador have recently been described by Arteaga et al. (2016). They identified several geographical barriers to dispersion, especially river drainages, and their data suggests that diversification also follows thermal elevation gradients between the Chocoan region and the Andes. The speciation pattern in the *Oophaga* clade, described by Posso‐Terranova and Andrés (2016b), suggests that climatic gradients acted as a strong evolutionary force toward diversification. Although we cannot here address the role of a climatic gradient in diversification events, the sPCA analysis (Figure 6) does not support the role of isolation by distance and several natural barriers could likely explain the genetic pattern in *O. sylvatica*. For instance, three river drainages separate the landscape, following the genetic clustering of our populations. The main river drainage composed of the Esmeraldas and Guayllabamba Rivers has been identified in the geographical pattern of differentiation in other frog species, such as *Pristimantis nietoi* and *P. walkeri*, populations of the snake *Bothrops punctatus* (Arteaga et al., 2016), and roughly separates the distribution range of *O. sylvatica* in two main groups (Figures 2 and 6). The northern genetic cluster (i.e., San Antonio, Lita, Alto Tambo, Durango, and Otokiki) and two populations from the southern genetic cluster (i.e., Felfa and Cristóbal Colón) are located north of Esmeraldas River, with the rest of the southern cluster (i.e., Simón Bolívar, Puerto Quito, Cube, Quingüe, Santo Domingo, and La Maná) in the south. Within the northern part of the *O. sylvatica* range, the Mira River drainage separates the San Antonio population from Lita, Alto Tambo, Durango, Otokiki, distributed between the Mira and the Santiago Rivers (Figure 6), potentially explaining the genetic differentiation of the San Antonio population within the northern cluster. The Felfa and Cristóbal Colón populations show the most contrasting structure. While genetically clustering with the southern group, they have intermediate distributions between the main river drainage of Esmeraldas and the Santiago River, separating them from the rest of the northern and southern genetic clusters. Both populations have admixed proportions of the two lineages with a higher proportion of the southern cluster (Figure 4), but mitochondrial data suggest Felfa's ancestry is nested in the northern cluster (Figure 2). A likely scenario would suggest that founder individuals dispersed from the northern cluster across the Santiago River and established first the Felfa population. Diversification was then slowed by the large Esmeraldas River drainage while giving rise to the population of Cristóbal Colón. Finally, the southern cluster may have arisen from founder individuals passing the Esmeraldas River and rapidly radiating in the south and west. It is interesting to note that similar pattern of genetic differentiation and distribution of color morphotypes has been described for the main studied species of *Oophaga*,* O. granulifera*, and *O. pumilio* (Brusa et al., 2013; Hauswaldt et al., 2011; Wang & Shaffer, 2008). Each study identified a river drainage acting as a natural barrier between a northern and a southern genetic lineage. Dendrobatidae are terrestrial frogs with poor swimming skills, and the presence of river drainage, which can likely change their course and strength over time in such regions with extreme level of precipitations, can likely explain the complex pattern of genetic structure observed across many species.

Although we now have a better understanding of population structure in *O. sylvatica*, a critical question is raised from this study: How is high phenotypic diversity in coloration and patterning maintained in the Otokiki population? A wide range of putative predators such as birds, reptiles, or arthropods with distinct visual abilities and predatory strategies might act on color selection and diversity among *O. sylvatica* populations (Crothers & Cummings, 2013; Dreher et al., 2015). In addition, sexual selection through nonrandom courtship within color morphs has been reported in *O. pumilio* (Reynolds & Fitzpatrick, 2007) and *Ranitomeya* (Twomey et al., 2014) and can possibly promote diversification of color among populations, while decreasing the variation within. However, this phenomenon seems highly dependent on the environmental context and genetic background of the frogs (Medina et al., 2013; Meuche, Brusa, Linsenmair, Keller, & Pröhl, 2013; Richards‐Zawacki et al., 2012; Twomey et al., 2014). A recent study reported an example of hybridization promoting new coloration and patterning between two close species *O. histrionica* and *O. lehmanni* (Medina et al., 2013). This work also suggests that complex roles are played by sexual selection, as hybrid females present nonrandom sexual preferences depending on the combination of available males. If similar processes occur in *O. sylvatica* at Otokiki, the extreme polymorphism observed in this locality represents a unique opportunity to further test the balance of natural and sexual selective pressures on the evolution of an aposematic trait.

### 4.1 Summary

By evaluating mitochondrial DNA variation and genome‐wide SNPs, we have gained four important insights about *O. sylvatica*: (1) The Ecuadorian populations of *O. sylvatica* are composed of two clades that reflect their geographic distribution. Further behavioral, ecological, and morphological information is required to determine whether the two geographical lineages observed in *Oophaga sylvatica* represent one polymorphic or several species. A combination of climatic gradient and structured landscape generating geographic barriers to gene flow could explain the complex patterns of diversification observed in *Oophaga* and some other Dendrobatidae (Arteaga et al., 2016; Posso‐Terranova & Andrés, 2016a, 2016b). (2) The northern and southern populations show different amounts of structure, which may reflect more recent range expansions in the south. (3) Phenotypic variation in *O. sylvatica* has evolved faster than genome‐wide mutations can fix in the population, suggesting that polymorphism is restricted to a small number of genes. (4) A highly polymorphic population (Otokiki) exists, which provides a unique opportunity for testing hypotheses about the selective pressures shaping aposematic traits. We hypothesize that this polymorphic population arose from either gene flow between phenotypically divergent populations at secondary contact zones or through a range expansion of the polymorphic Otokiki population into surrounding regions. More data are needed to distinguish between these alternative scenarios.

## DATA ARCHIVING

Sequence data have been submitted to GenBank: accession numbers 12S: KX553997–KX554204, 16S: KX554205–KX554413, CO1: KX574018–KX574226, NCX: KX785882–KX786090, RAG1: KX785673–KX785881, and TYR: KX785464–KX785672.

Raw data from ddRADseq reads have been submitted to SRA: SRP078453.

## AUTHOR CONTRIBUTIONS

ABR, JCS, LAC, and LAO designed the research; ABR, JCS, EET, LAC, and LAO collected samples in the field; ABR, BCC, and SNC performed the laboratory research; ABR analyzed data; ABR and LAO wrote the paper with contributions from all authors.

## CONFLICT OF INTEREST

The authors declare no conflict of interest.
