# Supplementary figures and images for: Radiation of the polymorphic Little Devil poison frog (Oophaga sylvatica) in Ecuador
Source: Ecol Evol. 2017 Oct 18;7(22):9750–62. doi: 10.1002/ece3.3503 (PMC5696431; doi:10.1002/ece3.3503)

Felfa

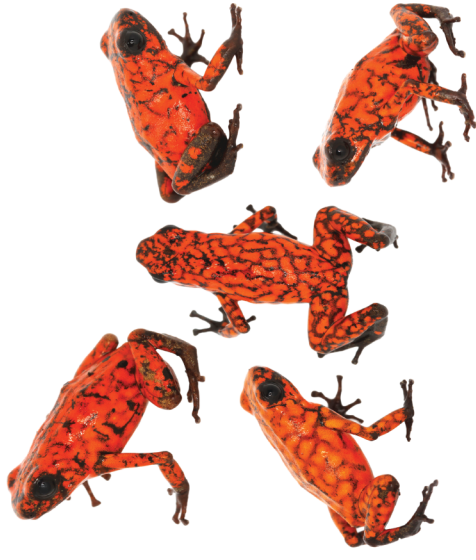

Puerto Quito

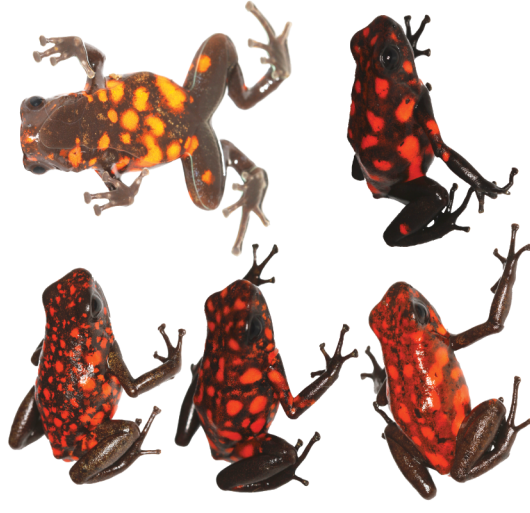

Lita

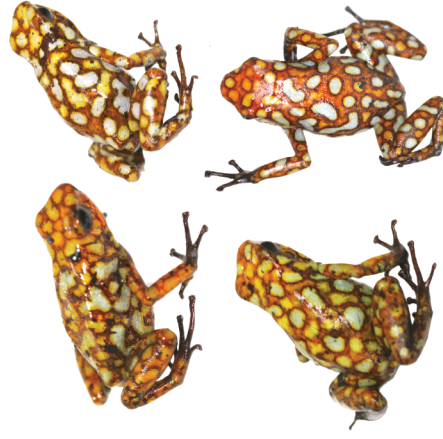

Durango

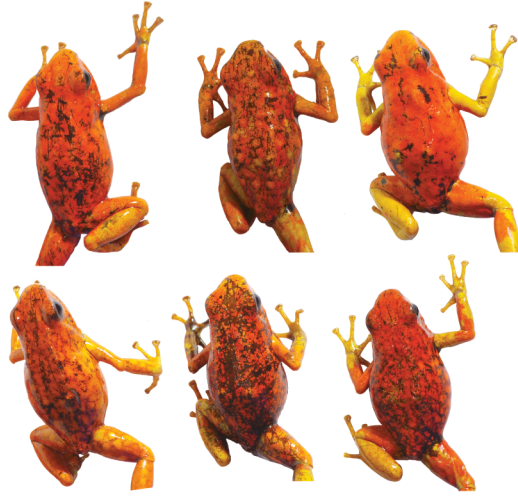

Cristóbal Colón

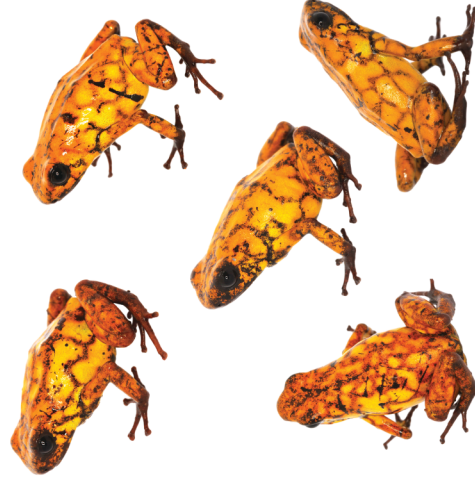

Supplement: Supplementary file 1 [file ECE3-7-9750-s001.pdf]

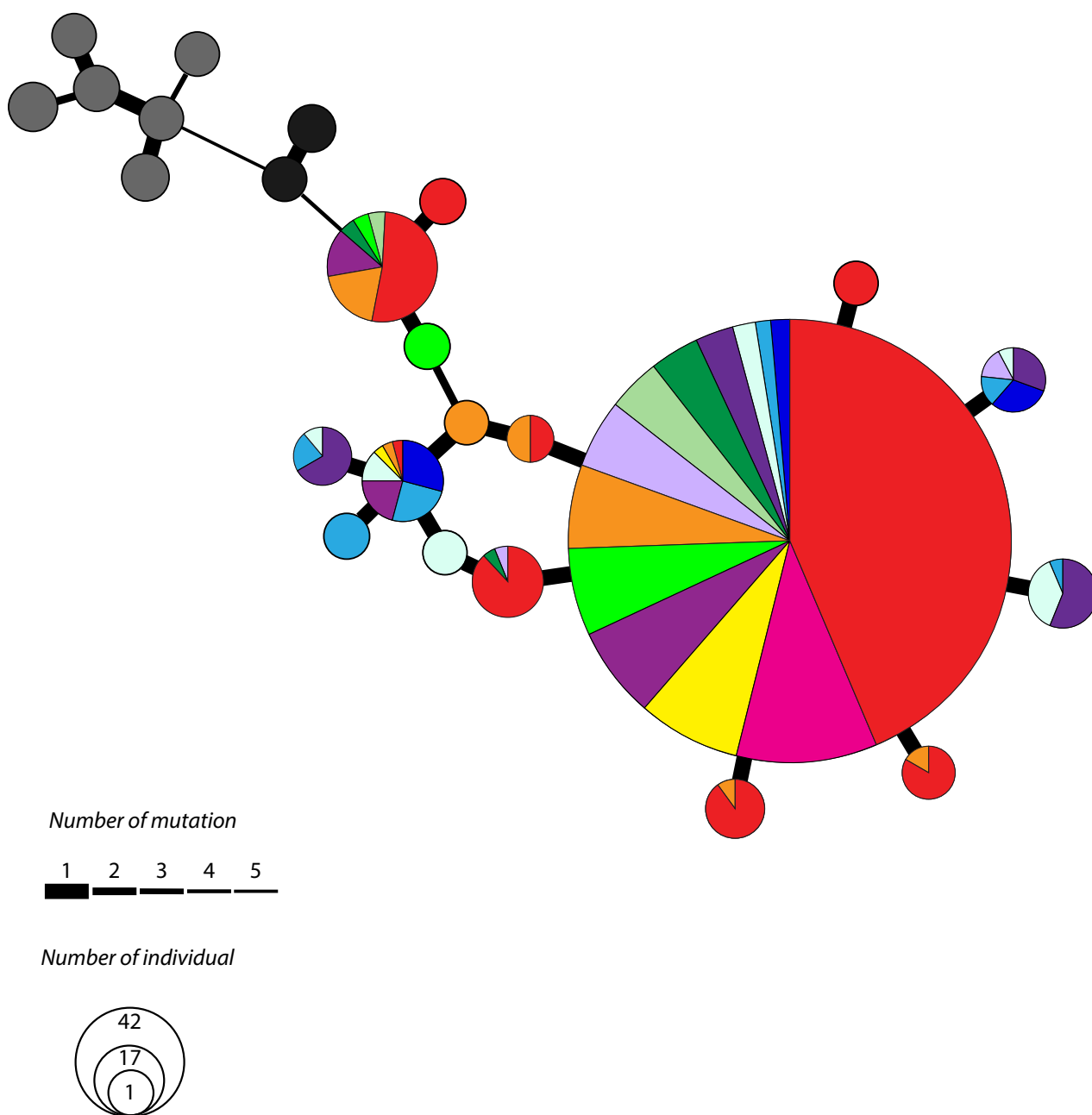

Figure 4

Supplement: Supplementary file 2 [file ECE3-7-9750-s002.pdf]

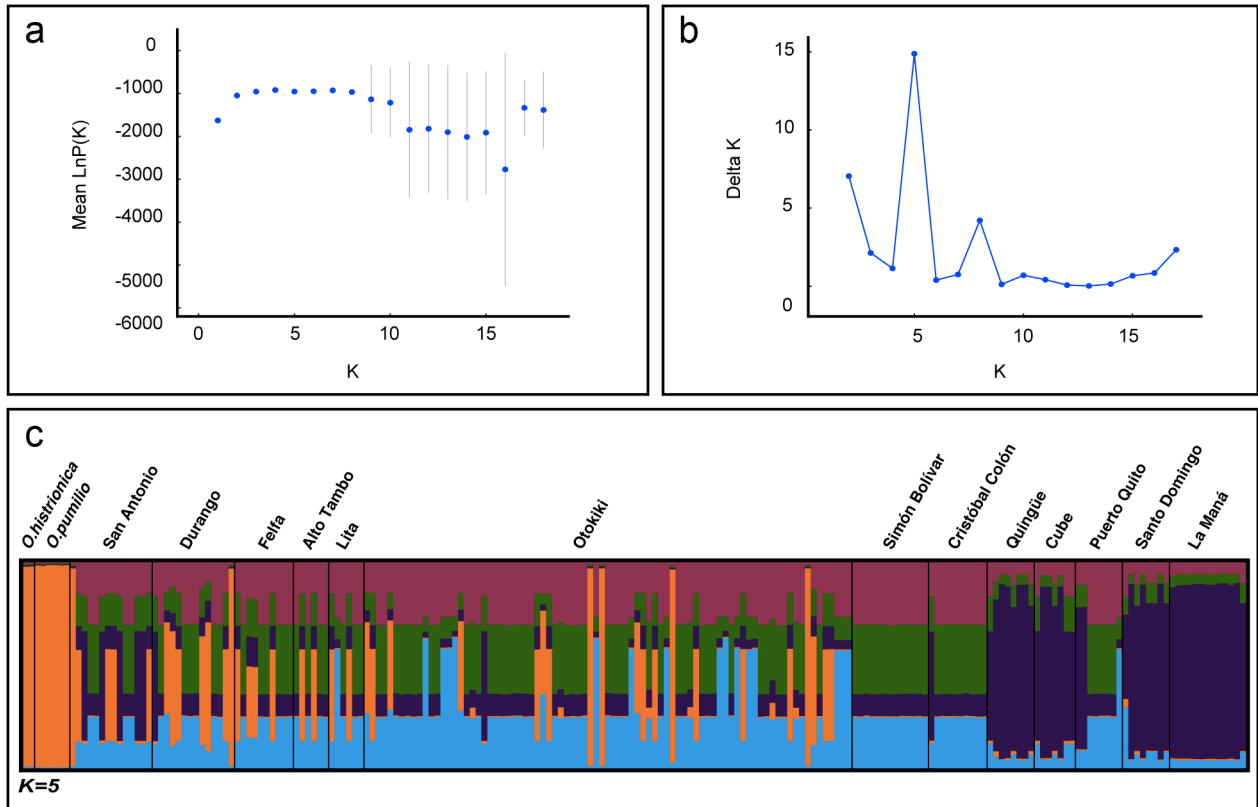

Suppl. Fig. 3

Supplement: Supplementary file 3 [file ECE3-7-9750-s003.pdf]

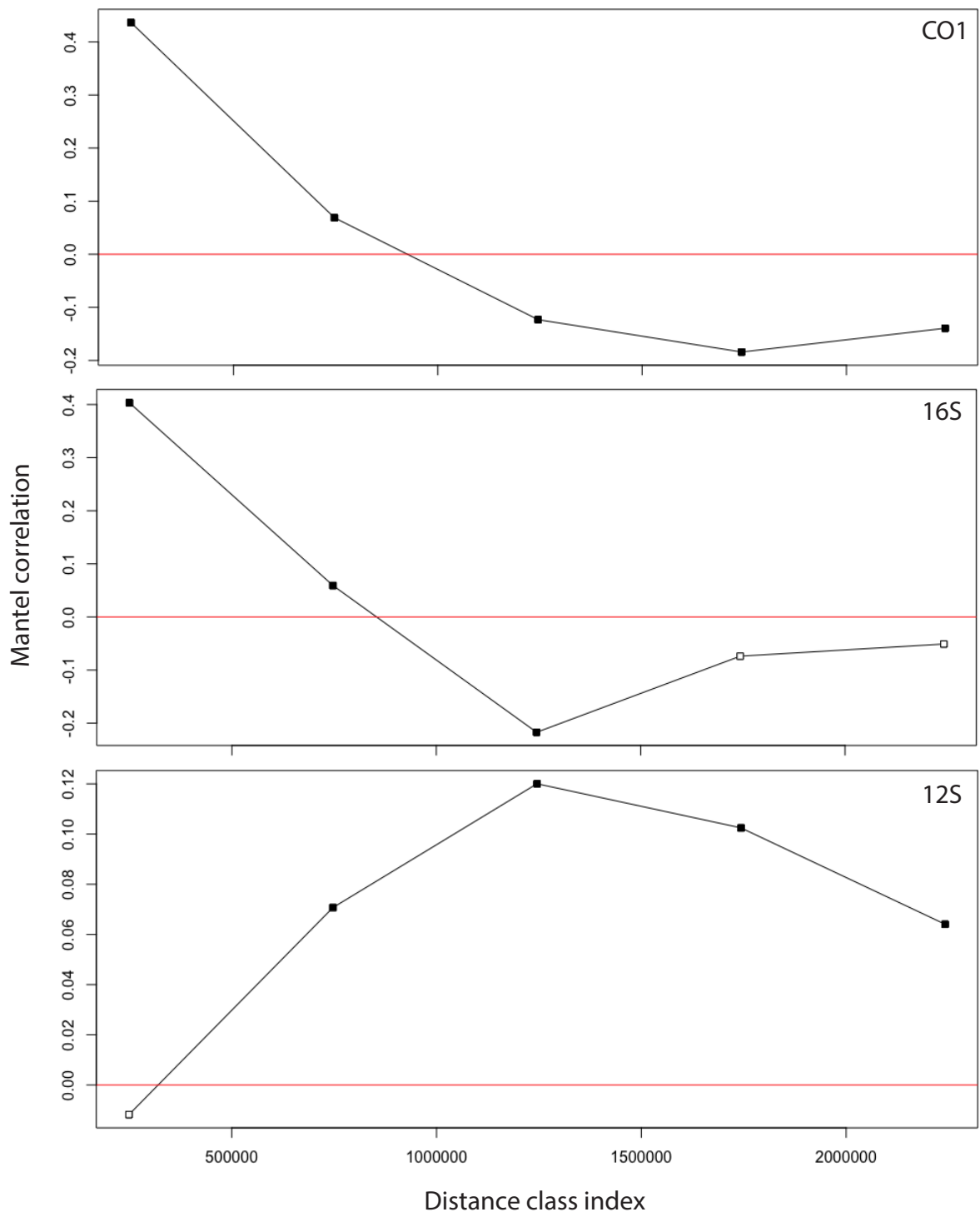

Supplement: Supplementary file 4 [file ECE3-7-9750-s004.pdf]
