# Supplementary material for: Radiation of the polymorphic Little Devil poison frog (Oophaga sylvatica) in Ecuador
Source: Ecol Evol. 2017 Oct 18;7(22):9750–62. doi: 10.1002/ece3.3503 (PMC5696431; doi:10.1002/ece3.3503)
Supplement: Supplementary file 5 [file ECE3-7-9750-s005.docx]

**Supplementary Table 1**

Identification key of the *O. sylvatica* samples from PCR amplicons and ddRAD sequencing.

| **Voucher ID** | **Population** | **Voucher ID** | **Population** | **Voucher ID** | **Population** | **Voucher ID** | | **Population** |
| --- | --- | --- | --- | --- | --- | --- | --- | --- |
| CJ1513 | Otokiki | CJ1660 | Otokiki | CJ1727 | Cube | CJ1926 | San Antonio | |
| CJ1514 | Otokiki | CJ1661 | Otokiki | CJ1728 | Cube | CJ1930 | San Antonio | |
| CJ1515 | Otokiki | CJ1662 | Otokiki | CJ1729 | La Maná | CJ1932 | San Antonio | |
| CJ1516 | Otokiki | CJ1663 | Otokiki | CJ1730 | La Maná | CJ1934 | San Antonio | |
| CJ1517 | Otokiki | CJ1664 | Otokiki | CJ1731 | La Maná | CJ1935 | San Antonio | |
| CJ1518 | Otokiki | CJ1665 | Otokiki | CJ1732 | La Maná | CJ1936 | San Antonio | |
| CJ1519 | Otokiki | CJ1666 | Otokiki | CJ1733 | La Maná | CJ2089 | Alto Tambo | |
| CJ1520 | Otokiki | CJ1667 | Otokiki | CJ1735 | La Maná | CJ2093 | Durango | |
| CJ1521 | Otokiki | CJ1668 | Otokiki | CJ1737 | La Maná | CJ2094 | Durango | |
| CJ1522 | Otokiki | CJ1669 | Otokiki | CJ1738 | La Maná | CJ2096 | Durango | |
| CJ1523 | Otokiki | CJ1670 | Otokiki | CJ1739 | La Maná | CJ2100 | Durango | |
| CJ1524 | Otokiki | CJ1671 | Otokiki | CJ1740 | La Maná | CJ2101 | Durango | |
| CJ1526 | Otokiki | CJ1672 | Otokiki | CJ1741 | La Maná | CJ2103 | Durango | |
| CJ1527 | Otokiki | CJ1673 | Otokiki | CJ1744 | La Maná | CJ2104 | Durango | |
| CJ1528 | Otokiki | CJ1674 | Otokiki | CJ1745 | La Maná | CJ2105 | Durango | |
| CJ1529 | Otokiki | CJ1675 | Otokiki | CJ1749 | Santo Domingo | CJ3089 | Cristóbal Colón | |
| CJ1530 | Otokiki | CJ1676 | Otokiki | CJ1750 | Santo Domingo | CJ3090 | Cristóbal Colón | |
| CJ1531 | Otokiki | CJ1677 | Otokiki | CJ1751 | Santo Domingo | CJ3091 | Cristóbal Colón | |
| CJ1532 | Otokiki | CJ1678 | Otokiki | CJ1752 | Santo Domingo | CJ3092 | Cristóbal Colón | |
| CJ1533 | Otokiki | CJ1679 | Otokiki | CJ1753 | Santo Domingo | CJ3093 | Cristóbal Colón | |
| CJ1534 | Otokiki | CJ1680 | Otokiki | CJ1756 | Santo Domingo | CJ3094 | Cristóbal Colón | |
| CJ1535 | Otokiki | CJ1681 | Otokiki | CJ1759 | Santo Domingo | CJ3095 | Cristóbal Colón | |
| CJ1631 | Otokiki | CJ1682 | Otokiki | CJ1760 | Santo Domingo | CJ3096 | Cristóbal Colón | |
| CJ1632 | Otokiki | CJ1683 | Otokiki | CJ1761 | Puerto Quito | CJ3097 | Cristóbal Colón | |
| CJ1633 | Otokiki | CJ1684 | Otokiki | CJ1762 | Puerto Quito | CJ3099 | Cristóbal Colón | |
| CJ1634 | Otokiki | CJ1685 | Otokiki | CJ1763 | Puerto Quito | CJ3105 | Simón Bolívar | |
| CJ1635 | Otokiki | CJ1687 | Otokiki | CJ1764 | Puerto Quito | CJ3106 | Simón Bolívar | |
| CJ1636 | Otokiki | CJ1688 | Otokiki | CJ1765 | Puerto Quito | CJ3107 | Simón Bolívar | |
| CJ1637 | Otokiki | CJ1690 | Otokiki | CJ1766 | Puerto Quito | CJ3108 | Simón Bolívar | |
| CJ1638 | Otokiki | CJ1691 | Otokiki | CJ1767 | Puerto Quito | CJ3109 | Simón Bolívar | |
| CJ1639 | Otokiki | CJ1692 | Otokiki | CJ1768 | Puerto Quito | CJ3110 | Simón Bolívar | |
| CJ1640 | Otokiki | CJ1693 | Otokiki | CJ1770 | Alto Tambo | CJ3111 | Simón Bolívar | |
| CJ1641 | Otokiki | CJ1694 | Lita | CJ1771 | Alto Tambo | CJ3112 | Simón Bolívar | |
| CJ1642 | Otokiki | CJ1695 | Lita | CJ1772 | Alto Tambo | CJ3113 | Simón Bolívar | |
| CJ1643 | Otokiki | CJ1696 | Lita | CJ1773 | Alto Tambo | CJ3114 | Simón Bolívar | |
| CJ1644 | Otokiki | CJ1697 | Lita | CJ1781 | Durango | CJ3115 | Simón Bolívar | |
| CJ1645 | Otokiki | CJ1698 | Lita | CJ1782 | Durango | CJ3116 | Simón Bolívar | |
| CJ1646 | Otokiki | CJ1699 | Lita | CJ1783 | Durango | CJ3123 | Simón Bolívar | |
| CJ1647 | Otokiki | CJ1702 | Quingüe | CJ1785 | Durango | CJ3124 | Felfa | |
| CJ1648 | Otokiki | CJ1703 | Quingüe | CJ1786 | Durango | CJ3125 | Felfa | |
| CJ1649 | Otokiki | CJ1704 | Quingüe | CJ1788 | Durango | CJ3126 | Felfa | |
| CJ1650 | Otokiki | CJ1705 | Quingüe | CJ1789 | Alto Tambo | CJ3127 | Felfa | |
| CJ1651 | Otokiki | CJ1708 | Quingüe | CJ1912 | San Antonio | CJ3128 | Felfa | |
| CJ1652 | Otokiki | CJ1709 | Quingüe | CJ1914 | San Antonio | CJ3129 | Felfa | |
| CJ1653 | Otokiki | CJ1712 | Quingüe | CJ1915 | San Antonio | CJ3130 | Felfa | |
| CJ1654 | Otokiki | CJ1713 | Quingüe | CJ1917 | San Antonio | CJ3131 | Felfa | |
| CJ1655 | Otokiki | CJ1715 | Cube | CJ1918 | San Antonio | CJ3132 | Felfa | |
| CJ1656 | Otokiki | CJ1716 | Cube | CJ1919 | San Antonio | CJ3133 | Felfa | |
| CJ1657 | Otokiki | CJ1719 | Cube | CJ1921 | San Antonio |  |  | |
| CJ1658 | Otokiki | CJ1720 | Cube | CJ1922 | San Antonio |  |  | |
| CJ1659 | Otokiki | CJ1722 | Cube | CJ1923 | San Antonio |  |  | |
