# Supplementary material for: Radiation of the polymorphic Little Devil poison frog (Oophaga sylvatica) in Ecuador
Source: Ecol Evol. 2017 Oct 18;7(22):9750–62. doi: 10.1002/ece3.3503 (PMC5696431; doi:10.1002/ece3.3503)
Supplement: Supplementary file 6 [file ECE3-7-9750-s006.docx]

**Supplementary Table 2**

Summary of species and within-population diversity for each nuclear gene (RAG-1, TYR and NCX). n, number of individuals sequenced; S, number of segregating sites; H, number of haplotypes; Hd, haplotype diversity; K, sequence diversity; π, nucleotide diversity.

| Marker | Species/Population | | n | S | H | Hd | K | π |
| --- | --- | --- | --- | --- | --- | --- | --- | --- |
| NCX | *O.sylvatica* | | 400 | 7 | 12 | 0.48169 | 1.35703 | 0.00122 |
| (1112 bp) |  | Durango | 28 | 5 | 6 | 0.60847 | 2.25132 | 0.00202 |
|  |  | Lita | 12 | 5 | 2 | 0.30303 | 1.51515 | 0.00136 |
|  |  | Alto Tambo | 12 | 5 | 2 | 0.30303 | 1.51515 | 0.00136 |
|  |  | Otokiki | 166 | 5 | 6 | 0.32844 | 1.31114 | 0.00118 |
|  |  | San Antonio | 28 | 5 | 3 | 0.57407 | 1.89153 | 0.0017 |
|  |  | Felfa | 20 | 5 | 3 | 0.35789 | 1.53684 | 0.00138 |
|  |  | Quingüe | 16 | 3 | 5 | 0.70833 | 1.05833 | 0.00095 |
|  |  | Cube | 14 | 2 | 3 | 0.67033 | 0.97802 | 0.00088 |
|  |  | Cristóbal Colón | 20 | 1 | 2 | 0.1 | 0.1 | 0.00009 |
|  |  | Simón Bolívar | 26 | 0 | 1 | 0 | 0 | 0 |
|  |  | Puerto Quito | 16 | 1 | 2 | 0.23333 | 0.23333 | 0.00021 |
|  |  | Santo Domingo | 16 | 3 | 5 | 0.78333 | 1.10833 | 0.001 |
|  |  | La Maná | 26 | 3 | 5 | 0.77231 | 1.14769 | 0.00103 |
|  | *O. histrionica* | | 4 | 0 | 1 | 0 | 0 | 0 |
|  | *O. pumilio* | | 12 | 1 | 2 | 0.16667 | 0.16667 | 0.00015 |
|  | Overall | | 416 | 10 | 15 | 0.52013 | 1.64247 | 0.00148 |
| RAG-1 | *O.sylvatica* | | 400 | 2 | 3 | 0.10471 | 0.10521 | 0.00015 |
| (705 bp) |  | Durango | 28 | 0 | 1 | 0 | 0 | 0 |
|  |  | Lita | 12 | 1 | 2 | 0.16667 | 0.16667 | 0.00024 |
|  |  | Alto Tambo | 12 | 0 | 1 | 0 | 0 | 0 |
|  |  | Otokiki | 166 | 1 | 2 | 0.18496 | 0.18496 | 0.00026 |
|  |  | San Antonio | 28 | 0 | 1 | 0 | 0 | 0 |
|  |  | Felfa | 20 | 0 | 1 | 0 | 0 | 0 |
|  |  | Quingüe | 16 | 1 | 2 | 0.23333 | 0.23333 | 0.00033 |
|  |  | Cube | 14 | 0 | 1 | 0 | 0 | 0 |
|  |  | Cristóbal Colón | 20 | 0 | 1 | 0 | 0 | 0 |
|  |  | Simón Bolívar | 26 | 0 | 1 | 0 | 0 | 0 |
|  |  | Puerto Quito | 16 | 1 | 2 | 0.125 | 0.125 | 0.00018 |
|  |  | Santo Domingo | 16 | 1 | 2 | 0.125 | 0.125 | 0.00018 |
|  |  | La Maná | 26 | 0 | 1 | 0 | 0 | 0 |
|  | *O. histrionica* | | 4 | 0 | 1 | 0 | 0 | 0 |
|  | *O. pumilio* | | 12 | 1 | 2 | 0.48485 | 0.48485 | 0.00069 |
|  | Overall | | 416 | 4 | 5 | 0.13684 | 0.13953 | 0.0002 |
| TYR | *O.sylvatica* | | 400 | 1 | 2 | 0.04887 | 0.04887 | 0.00011 |
| (438 bp) |  | Durango | 28 | 1 | 2 | 0.07143 | 0.07143 | 0.00016 |
|  |  | Lita | 12 | 0 | 1 | 0 | 0 | 0 |
|  |  | Alto Tambo | 12 | 0 | 1 | 0 | 0 | 0 |
|  |  | Otokiki | 166 | 1 | 2 | 0.10318 | 0.10318 | 0.00024 |
|  |  | San Antonio | 28 | 0 | 1 | 0 | 0 | 0 |
|  |  | Felfa | 20 | 0 | 1 | 0 | 0 | 0 |
|  |  | Quingüe | 16 | 0 | 1 | 0 | 0 | 0 |
|  |  | Cube | 14 | 0 | 1 | 0 | 0 | 0 |
|  |  | Cristóbal Colón | 20 | 0 | 1 | 0 | 0 | 0 |
|  |  | Simón Bolívar | 26 | 0 | 1 | 0 | 0 | 0 |
|  |  | Puerto Quito | 16 | 0 | 1 | 0 | 0 | 0 |
|  |  | Santo Domingo | 16 | 0 | 1 | 0 | 0 | 0 |
|  |  | La Maná | 26 | 0 | 1 | 0 | 0 | 0 |
|  | *O. histrionica* | | 4 | 1 | 2 | 0.5 | 0.5 | 0.00114 |
|  | *O. pumilio* | | 12 | 5 | 5 | 0.80303 | 1.56061 | 0.00356 |
|  | Overall | | 416 | 9 | 9 | 0.12053 | 0.28218 | 0.00064 |
