# Supplementary material for: Radiation of the polymorphic Little Devil poison frog (Oophaga sylvatica) in Ecuador
Source: Ecol Evol. 2017 Oct 18;7(22):9750–62. doi: 10.1002/ece3.3503 (PMC5696431; doi:10.1002/ece3.3503)
Supplement: Supplementary file 7 [file ECE3-7-9750-s007.docx]

**Supplementary Table 3**

Differentiation between population of *Oophaga sylvatica* by pairwise F_ST_ (upper diagonal), pairwise ϕ_ST_ (lower diagonal) and within population (diagonal values in grey) for concatenated mitochondrial genes. Values that are non-significant (p > 0.05) after 10,000 permutations are marked with a star and italicized.

| F_ST_  Φ_ST_ | San Antonio | Durango | Lita | Alto Tambo | Otokiki | Felfa | Cristóbal Colón | Simón Bolívar | Puerto Quito | Cube | Quingüe | Santo Domingo | La Maná |
| --- | --- | --- | --- | --- | --- | --- | --- | --- | --- | --- | --- | --- | --- |
| San Antonio | 5.824 | 0.651 | 0.595 | 0.595 | 0.472 | 0.356 | 0.316 | 0.321 | 0.364 | 0.37 | 0.469 | 0.394 | 0.689 |
| Durango | 0.269 | 3.34 | 0.118 | **0.064* | 0.086 | 0.217 | 0.18 | 0.193 | 0.222 | 0.225 | 0.328 | 0.253 | 0.561 |
| Lita | 0.291 | 0.377 | 3.8 | **0.047* | **0.002* | 0.228 | 0.183 | 0.197 | 0.234 | 0.237 | 0.372 | 0.273 | 0.705 |
| Alto Tambo | 0.263 | 0.321 | 0.26 | 6.066 | **0.033* | 0.197 | 0.153 | 0.168 | 0.203 | 0.205 | 0.341 | 0.242 | 0.678 |
| Otokiki | 0.188 | 0.261 | **0.017* | **0.077* | 8.369 | 0.149 | 0.119 | 0.13 | 0.153 | 0.155 | 0.234 | 0.177 | 0.371 |
| Felfa | 0.674 | 0.784 | 0.748 | 0.719 | 0.527 | 3.833 | 0.263 | 0.272 | 0.313 | 0.318 | 0.434 | 0.348 | 0.705 |
| Cristóbal Colón | 0.708 | 0.827 | 0.809 | 0.786 | 0.582 | 0.814 | 2.156 | 0.233 | 0.269 | 0.344 | 0.374 | 0.303 | 0.651 |
| Simón Bolívar | 0.691 | 0.813 | 0.782 | 0.772 | 0.586 | 0.783 | 0.254 | 3 | **-0.011* | **0.095* | **0.057* | **0.035* | 0.298 |
| Puerto Quito | 0.691 | 0.828 | 0.809 | 0.779 | 0.569 | 0.804 | 0.293 | **0.016* | 1.857 | **0.209* | **0.238* | **0.064* | 0.416 |
| Cube | 0.694 | 0.847 | 0.849 | 0.811 | 0.583 | 0.831 | 0.273 | **0.028* | **0.058* | 0.762 | **0.129* | **0.010* | 0.348 |
| Quingüe | 0.714 | 0.858 | 0.868 | 0.831 | 0.588 | 0.847 | 0.386 | **0.077* | **0.114* | **0.046* | 0.429 | **0.025* | **0.227* |
| Santo Domingo | 0.71 | 0.853 | 0.859 | 0.823 | 0.587 | 0.84 | 0.357 | **0.102* | **0.219* | **0.105* | **0.114* | 0.679 | 0.28 |
| La Maná | 0.766 | 0.89 | 0.916 | 0.885 | 0.605 | 0.891 | 0.479 | **0.152* | 0.35 | 0.226 | **0.227* | 0.175 | 0 |
