# Supplementary material for: Radiation of the polymorphic Little Devil poison frog (Oophaga sylvatica) in Ecuador
Source: Ecol Evol. 2017 Oct 18;7(22):9750–62. doi: 10.1002/ece3.3503 (PMC5696431; doi:10.1002/ece3.3503)
Supplement: Supplementary file 8 [file ECE3-7-9750-s008.docx]

**Supplementary Table 4**

Mantel tests for each mitochondrial gene. Mantel R: Mantel coefficient; p-val1: one-tailed p-value (H_o_: R ≤ 0); p-val2: one-tailed p-value (H_o_: R ≥ 0); p-val3: two-tailed p-value (H_o_: R = 0); llim 2.5%: lower confidence limit; ulim: upper confidence limit. P-values were calculated after 20000 permutations.

| **Gene** | **Mantel R** | **p-val1** | **p-val2** | **p-val3** | **llim 2.5%** | **ulim 97.5%** |
| --- | --- | --- | --- | --- | --- | --- |
| CO1 | 0.684 | 5.10^-5^ | 1 | 5.10^-5^ | 0.606 | 0.713 |
| 16S | 0.635 | 5.10^-5^ | 1 | 5.10^-5^ | 0.536 | 0.666 |
| 12S | 0.584 | 5.10^-5^ | 1 | 5.10^-5^ | 0.407 | 0.628 |
