# Supplementary material for: Radiation of the polymorphic Little Devil poison frog (Oophaga sylvatica) in Ecuador
Source: Ecol Evol. 2017 Oct 18;7(22):9750–62. doi: 10.1002/ece3.3503 (PMC5696431; doi:10.1002/ece3.3503)
Supplement: Supplementary file 9 [file ECE3-7-9750-s009.docx]

**Supplementary Table 5**

Differentiation between population of *Oophaga sylvatica* by pairwise F_ST_ (upper diagonal) for the ddRAD data set composed of 3,785 SNPs.

| F_ST_ | Durango | Lita | Alto Tambo | Otokiki | Felfa | Cristóbal  Colón | Simón  Bolívar | Puerto Quito | Cube | Quingüe | Santo Domingo | La Maná |
| --- | --- | --- | --- | --- | --- | --- | --- | --- | --- | --- | --- | --- |
| San Antonio | 0.0163 | 0.0141 | 0.0151 | 0.0162 | 0.0303 | 0.0367 | 0.0480 | 0.0654 | 0.0449 | 0.0597 | 0.0671 | 0.0607 |
| Durango |  | 0.0101 | 0.0059 | 0.0063 | 0.0258 | 0.0353 | 0.0430 | 0.0552 | 0.0450 | 0.0579 | 0.0611 | 0.0546 |
| Lita |  |  | 0.0140 | 0.0072 | 0.0424 | 0.0569 | 0.0664 | 0.0840 | 0.0678 | 0.0937 | 0.0974 | 0.0876 |
| Alto  Tambo |  |  |  | 0.0082 | 0.0401 | 0.0512 | 0.0590 | 0.0826 | 0.0585 | 0.0950 | 0.0966 | 0.0823 |
| Otokiki |  |  |  |  | 0.0247 | 0.0314 | 0.0338 | 0.0330 | 0.0322 | 0.0300 | 0.0349 | 0.0327 |
| Felfa |  |  |  |  |  | 0.0295 | 0.0197 | 0.0466 | 0.0388 | 0.0724 | 0.0680 | 0.0498 |
| Cristóbal  Colón | |  |  |  |  |  | 0.0262 | 0.0636 | 0.0512 | 0.0832 | 0.0893 | 0.0658 |
| Simón  Bolívar | |  |  |  |  |  |  | 0.0552 | 0.0502 | 0.1030 | 0.0847 | 0.0597 |
| Puerto Quito |  |  |  |  |  |  |  |  | 0.0678 | 0.1370 | 0.0873 | 0.0894 |
| Cube |  |  |  |  |  |  |  |  |  | 0.0702 | 0.0537 | 0.0425 |
| Quingüe |  |  |  |  |  |  |  |  |  |  | 0.1222 | 0.0965 |
| Santo  Domingo | |  |  |  |  |  |  |  |  |  |  | 0.0546 |
