# Supplementary material for: Radiation of the polymorphic Little Devil poison frog (Oophaga sylvatica) in Ecuador
Source: Ecol Evol. 2017 Oct 18;7(22):9750–62. doi: 10.1002/ece3.3503 (PMC5696431; doi:10.1002/ece3.3503)
Supplement: Supplementary file 10 [file ECE3-7-9750-s010.pdf]

Ecology and Evolution

SUPPORTING INFORMATION

Radiation of the polymorphic Little Devil poison frog (*Oophaga sylvatica*) in Ecuador

Alexandre B. Roland[1], Juan C. Santos[2], Bella C. Carriker[3], Stephanie N. Caty[1], Elicio E. Tapia[4], Luis A. Coloma[4], and Lauren A. O'Connell[1*]

Appendix S1. Supplementary Figures

Supplementary Figure 1

Examples of variability in color patterning within monotypic populations

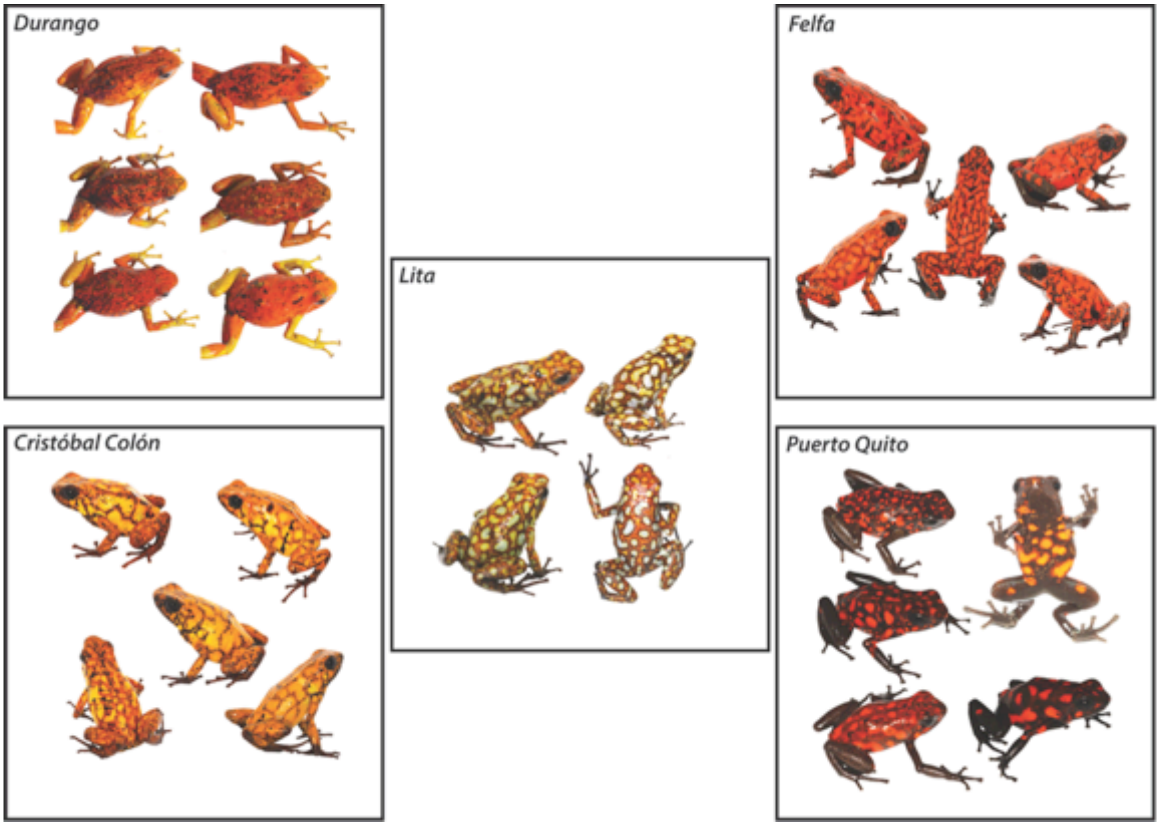

Supplementary Figure 2

Haplotype network of concatenated nuclear amplicons (NCX, RAG-1 and TYR) of *Oophaga sylvatica*, *O. histrionica* and *O. pumilio* (2255 bp). Circles indicate haplotypes, with the area being proportional to the number of individuals sharing that haplotype. Colours refer to the geographic origin of the population and the pie charts represent the percentage of each population sharing the same haplotype. Line thickness between haplotypes is proportional to the inferred mutational steps (or inferred intermediate haplotypes).

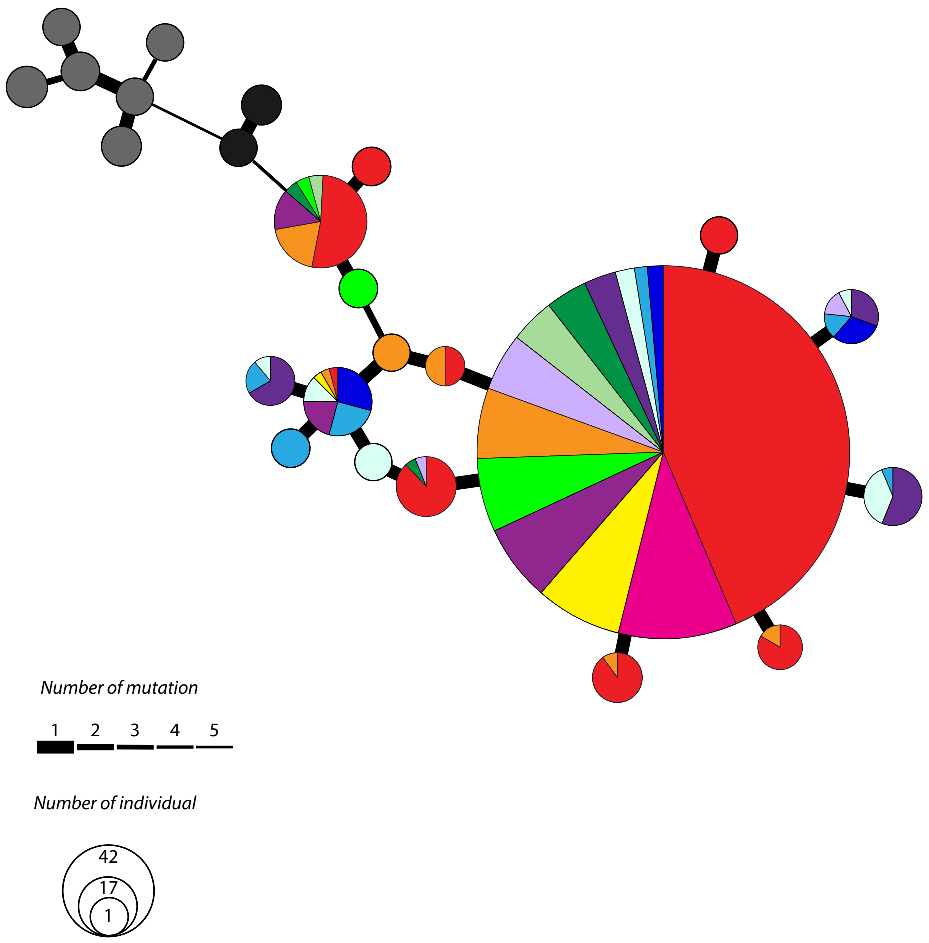

Supplementary Figure 3

Population structure inferred from concatenated nuclear amplicons (NCX, RAG-1 and TYR) for *Oophaga sylvatica*, *O. histrionica* and *O. pumilio* (2255 bp). a) Evolution of the mean LnP(K) in function of the number of putative K. b) Plot of ΔK values from 20 runs of STRUCTURE, varying K from 1 to 17. The most likely value for K was identified at K=5. c) Bar plots showing Bayesian assignment probabilities for individual frogs as inferred by STRUCTURE for five clusters, each color depicting one of the putative clusters.

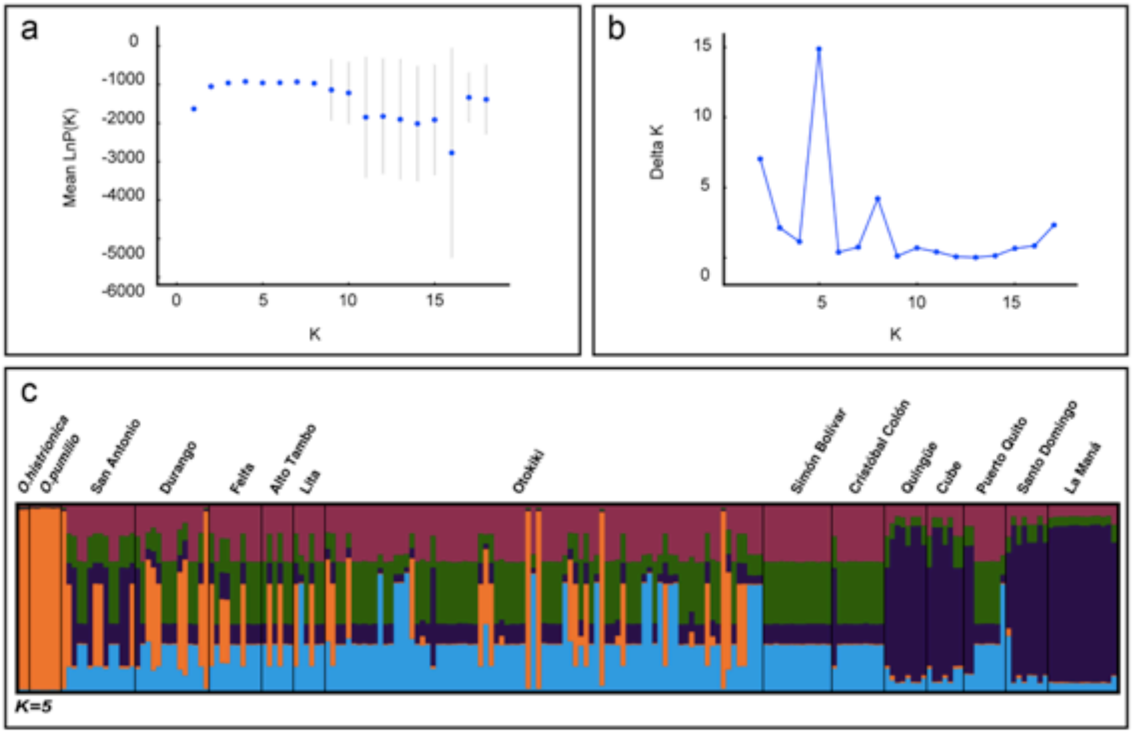

Supplementary Figure 4

Mantel correlogram for each mitochondrial gene.

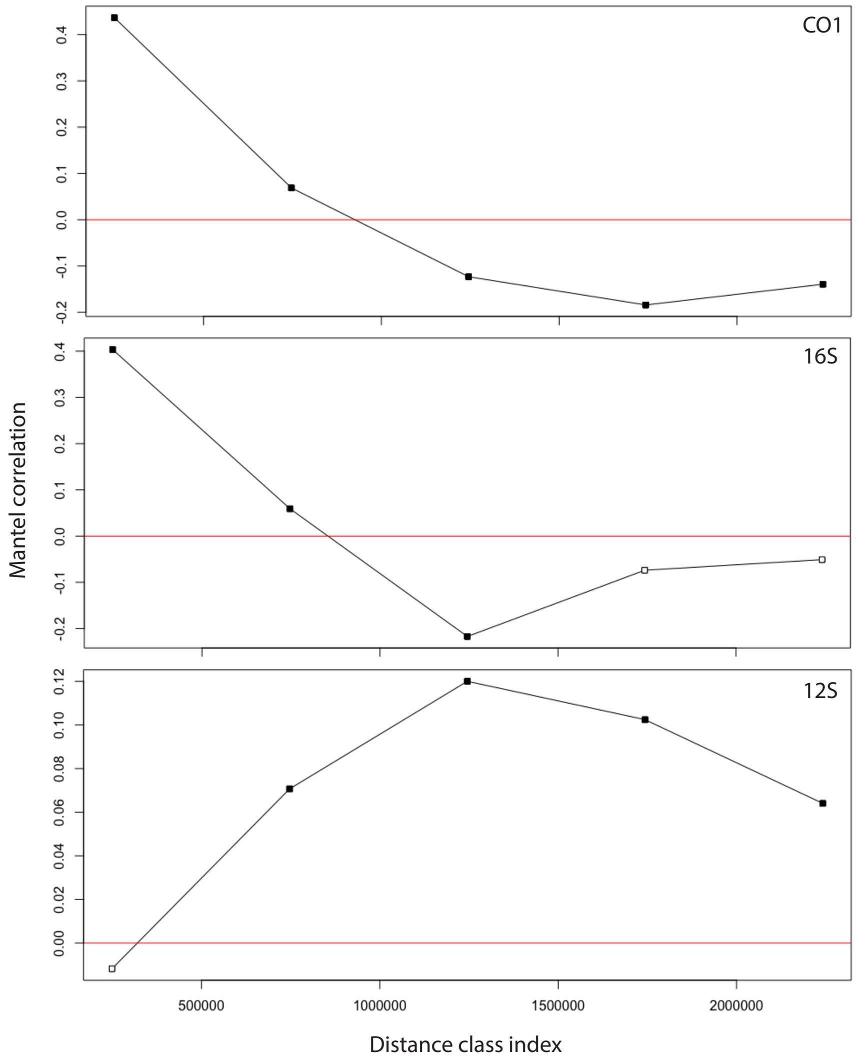

Supplementary Figure 5

Phylogeny of *O. sylvatica*. The best-fitting substitution models was estimated with JMODELTEST 2.1 and the General Time Reversible (GTR) model with gamma-distributed rate heterogeneity among the remaining sites (+G) was selected based on AIC, BIC and DT. The phylogeny was built using MR BAYES 3.2.6 in GENEIOUS, running a single MCMC with four chains (0.02 heated chain temp) for 1,100,000 generations, out of which the first 100,000 were discarded as burn-in, and *O. pumilio* was set as an outgroup. Numbers indicate the posterior probability of each node (> 0.7), and the color for individuals correspond to their original geographical population as defined in Figure 1a.

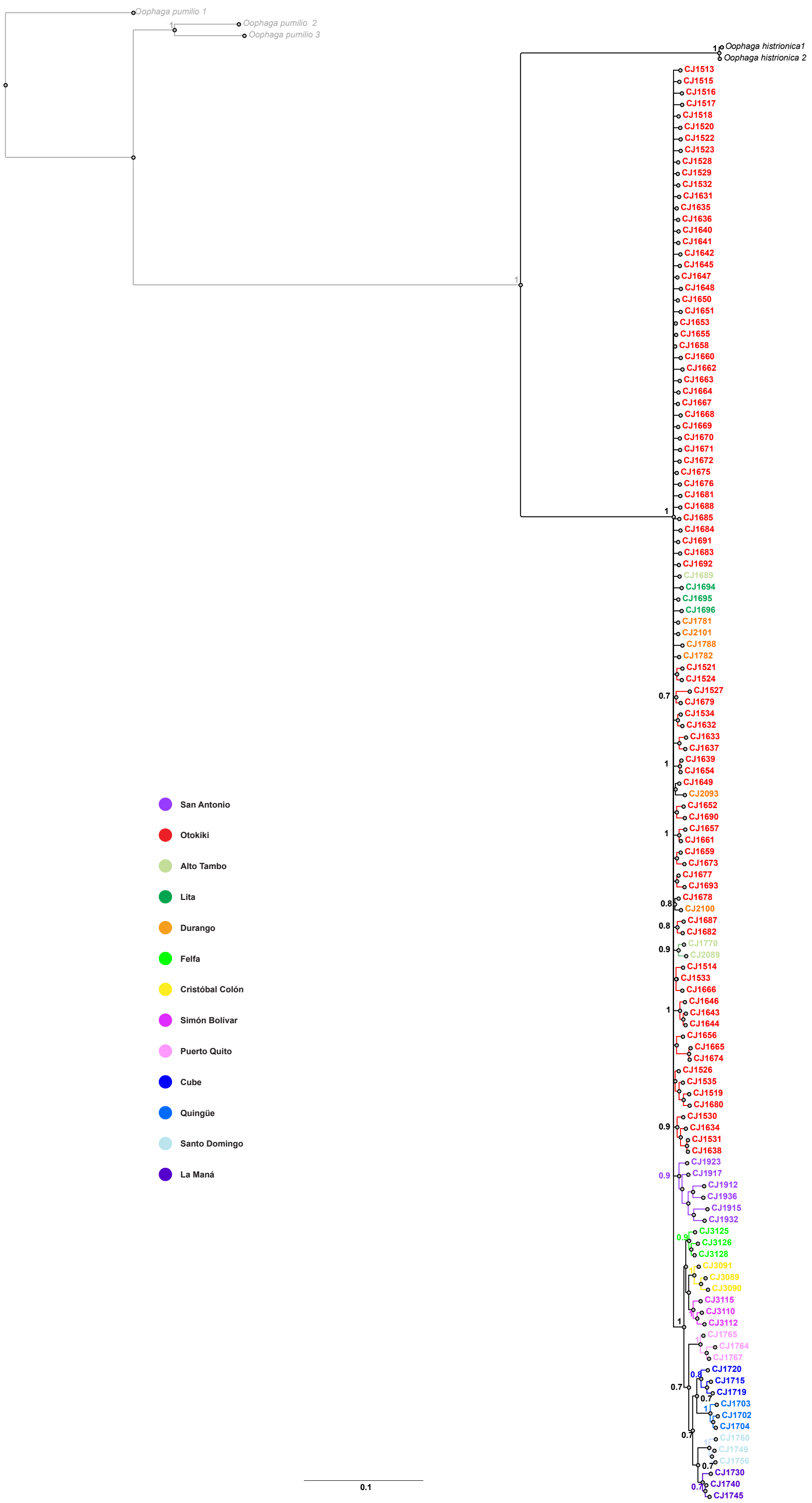
Oophaga pumilio 1
Oophaga pumilio 2
Oophaga pumilio 3
Oophaga histrionica1
Oophaga histrionica 2
CJ1513
CJ1515
CJ1516
CJ1517
CJ1518
CJ1520
CJ1522
CJ1523
CJ1528
CJ1529
CJ1532
CJ1631
CJ1635
CJ1636
CJ1640
CJ1641
CJ1642
CJ1645
CJ1647
CJ1648
CJ1650
CJ1651
CJ1653
CJ1655
CJ1658
CJ1660
CJ1662
CJ1663
CJ1664
CJ1667
CJ1668
CJ1669
CJ1670
CJ1671
CJ1672
CJ1675
CJ1676
CJ1681
CJ1688
CJ1685
CJ1684
CJ1691
CJ1683
CJ1692
CJ1689
CJ1694
CJ1695
CJ1696
CJ1781
CJ2101
CJ1788
CJ1782
CJ1521
CJ1524
CJ1527
CJ1679
CJ1534
CJ1632
CJ1633
CJ1637
CJ1639
CJ1654
CJ1649
CJ2093
CJ1652
CJ1690
CJ1657
CJ1661
CJ1659
CJ1673
CJ1677
CJ1693
CJ1678
CJ2100
CJ1687
CJ1682
CJ1770
CJ2089
CJ1514
CJ1533
CJ1666
CJ1646
CJ1643
CJ1644
CJ1656
CJ1665
CJ1674
CJ1526
CJ1535
CJ1519
CJ1680
CJ1530
CJ1634
CJ1531
CJ1638
CJ1923
CJ1917
CJ1912
CJ1936
CJ1915
CJ1932
CJ3125
CJ3126
CJ3128
CJ3091
CJ3089
CJ3090
CJ3115
CJ3110
CJ3112
CJ1765
CJ1764
CJ1767
CJ1720
CJ1715
CJ1719
CJ1703
CJ1702
CJ1704
CJ1760
CJ1749
CJ1756
CJ1730
CJ1740
CJ1745
San Antonio
Otokiki
Alto Tambo
Lita
Durango
Felfa
Cristóbal Colón
Simón Bolívar
Puerto Quito
Cube
Quingüe
Santo Domingo
La Maná
0.1
