# Supplementary material for: Radiation of the polymorphic Little Devil poison frog (Oophaga sylvatica) in Ecuador
Source: Ecol Evol. 2017 Oct 18;7(22):9750–62. doi: 10.1002/ece3.3503 (PMC5696431; doi:10.1002/ece3.3503)
Supplement: Supplementary file 11 [file ECE3-7-9750-s011.docx]

Ecology and Evolution

**SUPPORTING INFORMATION**

**Radiation of the polymorphic Little Devil poison frog (*Oophaga sylvatica*) in Ecuador**

Alexandre B. Roland^1^, Juan C. Santos^2^, Bella C. Carriker^3^, Stephanie N. Caty^1^, Elicio E. Tapia^4^, Luis A. Coloma^4^, and Lauren A. O’Connell^1*^

**Appendix S2. Supplementary Tables**

**Supplementary Table 1**

Identification key of the *O. sylvatica* samples from PCR amplicons and ddRAD sequencing.

| **Voucher ID** | **Population** | **Voucher ID** | **Population** | **Voucher ID** | **Population** | **Voucher ID** | | **Population** |
| --- | --- | --- | --- | --- | --- | --- | --- | --- |
| CJ1513 | Otokiki | CJ1660 | Otokiki | CJ1727 | Cube | CJ1926 | San Antonio | |
| CJ1514 | Otokiki | CJ1661 | Otokiki | CJ1728 | Cube | CJ1930 | San Antonio | |
| CJ1515 | Otokiki | CJ1662 | Otokiki | CJ1729 | La Maná | CJ1932 | San Antonio | |
| CJ1516 | Otokiki | CJ1663 | Otokiki | CJ1730 | La Maná | CJ1934 | San Antonio | |
| CJ1517 | Otokiki | CJ1664 | Otokiki | CJ1731 | La Maná | CJ1935 | San Antonio | |
| CJ1518 | Otokiki | CJ1665 | Otokiki | CJ1732 | La Maná | CJ1936 | San Antonio | |
| CJ1519 | Otokiki | CJ1666 | Otokiki | CJ1733 | La Maná | CJ2089 | Alto Tambo | |
| CJ1520 | Otokiki | CJ1667 | Otokiki | CJ1735 | La Maná | CJ2093 | Durango | |
| CJ1521 | Otokiki | CJ1668 | Otokiki | CJ1737 | La Maná | CJ2094 | Durango | |
| CJ1522 | Otokiki | CJ1669 | Otokiki | CJ1738 | La Maná | CJ2096 | Durango | |
| CJ1523 | Otokiki | CJ1670 | Otokiki | CJ1739 | La Maná | CJ2100 | Durango | |
| CJ1524 | Otokiki | CJ1671 | Otokiki | CJ1740 | La Maná | CJ2101 | Durango | |
| CJ1526 | Otokiki | CJ1672 | Otokiki | CJ1741 | La Maná | CJ2103 | Durango | |
| CJ1527 | Otokiki | CJ1673 | Otokiki | CJ1744 | La Maná | CJ2104 | Durango | |
| CJ1528 | Otokiki | CJ1674 | Otokiki | CJ1745 | La Maná | CJ2105 | Durango | |
| CJ1529 | Otokiki | CJ1675 | Otokiki | CJ1749 | Santo Domingo | CJ3089 | Cristóbal Colón | |
| CJ1530 | Otokiki | CJ1676 | Otokiki | CJ1750 | Santo Domingo | CJ3090 | Cristóbal Colón | |
| CJ1531 | Otokiki | CJ1677 | Otokiki | CJ1751 | Santo Domingo | CJ3091 | Cristóbal Colón | |
| CJ1532 | Otokiki | CJ1678 | Otokiki | CJ1752 | Santo Domingo | CJ3092 | Cristóbal Colón | |
| CJ1533 | Otokiki | CJ1679 | Otokiki | CJ1753 | Santo Domingo | CJ3093 | Cristóbal Colón | |
| CJ1534 | Otokiki | CJ1680 | Otokiki | CJ1756 | Santo Domingo | CJ3094 | Cristóbal Colón | |
| CJ1535 | Otokiki | CJ1681 | Otokiki | CJ1759 | Santo Domingo | CJ3095 | Cristóbal Colón | |
| CJ1631 | Otokiki | CJ1682 | Otokiki | CJ1760 | Santo Domingo | CJ3096 | Cristóbal Colón | |
| CJ1632 | Otokiki | CJ1683 | Otokiki | CJ1761 | Puerto Quito | CJ3097 | Cristóbal Colón | |
| CJ1633 | Otokiki | CJ1684 | Otokiki | CJ1762 | Puerto Quito | CJ3099 | Cristóbal Colón | |
| CJ1634 | Otokiki | CJ1685 | Otokiki | CJ1763 | Puerto Quito | CJ3105 | Simón Bolívar | |
| CJ1635 | Otokiki | CJ1687 | Otokiki | CJ1764 | Puerto Quito | CJ3106 | Simón Bolívar | |
| CJ1636 | Otokiki | CJ1688 | Otokiki | CJ1765 | Puerto Quito | CJ3107 | Simón Bolívar | |
| CJ1637 | Otokiki | CJ1690 | Otokiki | CJ1766 | Puerto Quito | CJ3108 | Simón Bolívar | |
| CJ1638 | Otokiki | CJ1691 | Otokiki | CJ1767 | Puerto Quito | CJ3109 | Simón Bolívar | |
| CJ1639 | Otokiki | CJ1692 | Otokiki | CJ1768 | Puerto Quito | CJ3110 | Simón Bolívar | |
| CJ1640 | Otokiki | CJ1693 | Otokiki | CJ1770 | Alto Tambo | CJ3111 | Simón Bolívar | |
| CJ1641 | Otokiki | CJ1694 | Lita | CJ1771 | Alto Tambo | CJ3112 | Simón Bolívar | |
| CJ1642 | Otokiki | CJ1695 | Lita | CJ1772 | Alto Tambo | CJ3113 | Simón Bolívar | |
| CJ1643 | Otokiki | CJ1696 | Lita | CJ1773 | Alto Tambo | CJ3114 | Simón Bolívar | |
| CJ1644 | Otokiki | CJ1697 | Lita | CJ1781 | Durango | CJ3115 | Simón Bolívar | |
| CJ1645 | Otokiki | CJ1698 | Lita | CJ1782 | Durango | CJ3116 | Simón Bolívar | |
| CJ1646 | Otokiki | CJ1699 | Lita | CJ1783 | Durango | CJ3123 | Simón Bolívar | |
| CJ1647 | Otokiki | CJ1702 | Quingüe | CJ1785 | Durango | CJ3124 | Felfa | |
| CJ1648 | Otokiki | CJ1703 | Quingüe | CJ1786 | Durango | CJ3125 | Felfa | |
| CJ1649 | Otokiki | CJ1704 | Quingüe | CJ1788 | Durango | CJ3126 | Felfa | |
| CJ1650 | Otokiki | CJ1705 | Quingüe | CJ1789 | Alto Tambo | CJ3127 | Felfa | |
| CJ1651 | Otokiki | CJ1708 | Quingüe | CJ1912 | San Antonio | CJ3128 | Felfa | |
| CJ1652 | Otokiki | CJ1709 | Quingüe | CJ1914 | San Antonio | CJ3129 | Felfa | |
| CJ1653 | Otokiki | CJ1712 | Quingüe | CJ1915 | San Antonio | CJ3130 | Felfa | |
| CJ1654 | Otokiki | CJ1713 | Quingüe | CJ1917 | San Antonio | CJ3131 | Felfa | |
| CJ1655 | Otokiki | CJ1715 | Cube | CJ1918 | San Antonio | CJ3132 | Felfa | |
| CJ1656 | Otokiki | CJ1716 | Cube | CJ1919 | San Antonio | CJ3133 | Felfa | |
| CJ1657 | Otokiki | CJ1719 | Cube | CJ1921 | San Antonio |  |  | |
| CJ1658 | Otokiki | CJ1720 | Cube | CJ1922 | San Antonio |  |  | |
| CJ1659 | Otokiki | CJ1722 | Cube | CJ1923 | San Antonio |  |  | |

**Supplementary Table 2**

Summary of species and within-population diversity for each nuclear gene (RAG-1, TYR and NCX). n, number of individuals sequenced; S, number of segregating sites; H, number of haplotypes; Hd, haplotype diversity; K, sequence diversity; π, nucleotide diversity.

| Marker | Species/Population | | n | S | H | Hd | K | π |
| --- | --- | --- | --- | --- | --- | --- | --- | --- |
| NCX | *O.sylvatica* | | 400 | 7 | 12 | 0.48169 | 1.35703 | 0.00122 |
| (1112 bp) |  | Durango | 28 | 5 | 6 | 0.60847 | 2.25132 | 0.00202 |
|  |  | Lita | 12 | 5 | 2 | 0.30303 | 1.51515 | 0.00136 |
|  |  | Alto Tambo | 12 | 5 | 2 | 0.30303 | 1.51515 | 0.00136 |
|  |  | Otokiki | 166 | 5 | 6 | 0.32844 | 1.31114 | 0.00118 |
|  |  | San Antonio | 28 | 5 | 3 | 0.57407 | 1.89153 | 0.0017 |
|  |  | Felfa | 20 | 5 | 3 | 0.35789 | 1.53684 | 0.00138 |
|  |  | Quingüe | 16 | 3 | 5 | 0.70833 | 1.05833 | 0.00095 |
|  |  | Cube | 14 | 2 | 3 | 0.67033 | 0.97802 | 0.00088 |
|  |  | Cristóbal Colón | 20 | 1 | 2 | 0.1 | 0.1 | 0.00009 |
|  |  | Simón Bolívar | 26 | 0 | 1 | 0 | 0 | 0 |
|  |  | Puerto Quito | 16 | 1 | 2 | 0.23333 | 0.23333 | 0.00021 |
|  |  | Santo Domingo | 16 | 3 | 5 | 0.78333 | 1.10833 | 0.001 |
|  |  | La Maná | 26 | 3 | 5 | 0.77231 | 1.14769 | 0.00103 |
|  | *O. histrionica* | | 4 | 0 | 1 | 0 | 0 | 0 |
|  | *O. pumilio* | | 12 | 1 | 2 | 0.16667 | 0.16667 | 0.00015 |
|  | Overall | | 416 | 10 | 15 | 0.52013 | 1.64247 | 0.00148 |
| RAG-1 | *O.sylvatica* | | 400 | 2 | 3 | 0.10471 | 0.10521 | 0.00015 |
| (705 bp) |  | Durango | 28 | 0 | 1 | 0 | 0 | 0 |
|  |  | Lita | 12 | 1 | 2 | 0.16667 | 0.16667 | 0.00024 |
|  |  | Alto Tambo | 12 | 0 | 1 | 0 | 0 | 0 |
|  |  | Otokiki | 166 | 1 | 2 | 0.18496 | 0.18496 | 0.00026 |
|  |  | San Antonio | 28 | 0 | 1 | 0 | 0 | 0 |
|  |  | Felfa | 20 | 0 | 1 | 0 | 0 | 0 |
|  |  | Quingüe | 16 | 1 | 2 | 0.23333 | 0.23333 | 0.00033 |
|  |  | Cube | 14 | 0 | 1 | 0 | 0 | 0 |
|  |  | Cristóbal Colón | 20 | 0 | 1 | 0 | 0 | 0 |
|  |  | Simón Bolívar | 26 | 0 | 1 | 0 | 0 | 0 |
|  |  | Puerto Quito | 16 | 1 | 2 | 0.125 | 0.125 | 0.00018 |
|  |  | Santo Domingo | 16 | 1 | 2 | 0.125 | 0.125 | 0.00018 |
|  |  | La Maná | 26 | 0 | 1 | 0 | 0 | 0 |
|  | *O. histrionica* | | 4 | 0 | 1 | 0 | 0 | 0 |
|  | *O. pumilio* | | 12 | 1 | 2 | 0.48485 | 0.48485 | 0.00069 |
|  | Overall | | 416 | 4 | 5 | 0.13684 | 0.13953 | 0.0002 |
| TYR | *O.sylvatica* | | 400 | 1 | 2 | 0.04887 | 0.04887 | 0.00011 |
| (438 bp) |  | Durango | 28 | 1 | 2 | 0.07143 | 0.07143 | 0.00016 |
|  |  | Lita | 12 | 0 | 1 | 0 | 0 | 0 |
|  |  | Alto Tambo | 12 | 0 | 1 | 0 | 0 | 0 |
|  |  | Otokiki | 166 | 1 | 2 | 0.10318 | 0.10318 | 0.00024 |
|  |  | San Antonio | 28 | 0 | 1 | 0 | 0 | 0 |
|  |  | Felfa | 20 | 0 | 1 | 0 | 0 | 0 |
|  |  | Quingüe | 16 | 0 | 1 | 0 | 0 | 0 |
|  |  | Cube | 14 | 0 | 1 | 0 | 0 | 0 |
|  |  | Cristóbal Colón | 20 | 0 | 1 | 0 | 0 | 0 |
|  |  | Simón Bolívar | 26 | 0 | 1 | 0 | 0 | 0 |
|  |  | Puerto Quito | 16 | 0 | 1 | 0 | 0 | 0 |
|  |  | Santo Domingo | 16 | 0 | 1 | 0 | 0 | 0 |
|  |  | La Maná | 26 | 0 | 1 | 0 | 0 | 0 |
|  | *O. histrionica* | | 4 | 1 | 2 | 0.5 | 0.5 | 0.00114 |
|  | *O. pumilio* | | 12 | 5 | 5 | 0.80303 | 1.56061 | 0.00356 |
|  | Overall | | 416 | 9 | 9 | 0.12053 | 0.28218 | 0.00064 |

**Supplementary Table 3**

Differentiation between population of *Oophaga sylvatica* by pairwise F_ST_ (upper diagonal), pairwise ϕ_ST_ (lower diagonal) and within population (diagonal values in grey) for concatenated mitochondrial genes. Values that are non-significant (p > 0.05) after 10,000 permutations are marked with a star and italicized.

| F_ST_  Φ_ST_ | San Antonio | Durango | Lita | Alto Tambo | Otokiki | Felfa | Cristóbal Colón | Simón Bolívar | Puerto Quito | Cube | Quingüe | Santo Domingo | La Maná |
| --- | --- | --- | --- | --- | --- | --- | --- | --- | --- | --- | --- | --- | --- |
| San Antonio | 5.824 | 0.651 | 0.595 | 0.595 | 0.472 | 0.356 | 0.316 | 0.321 | 0.364 | 0.37 | 0.469 | 0.394 | 0.689 |
| Durango | 0.269 | 3.34 | 0.118 | **0.064* | 0.086 | 0.217 | 0.18 | 0.193 | 0.222 | 0.225 | 0.328 | 0.253 | 0.561 |
| Lita | 0.291 | 0.377 | 3.8 | **0.047* | **0.002* | 0.228 | 0.183 | 0.197 | 0.234 | 0.237 | 0.372 | 0.273 | 0.705 |
| Alto Tambo | 0.263 | 0.321 | 0.26 | 6.066 | **0.033* | 0.197 | 0.153 | 0.168 | 0.203 | 0.205 | 0.341 | 0.242 | 0.678 |
| Otokiki | 0.188 | 0.261 | **0.017* | **0.077* | 8.369 | 0.149 | 0.119 | 0.13 | 0.153 | 0.155 | 0.234 | 0.177 | 0.371 |
| Felfa | 0.674 | 0.784 | 0.748 | 0.719 | 0.527 | 3.833 | 0.263 | 0.272 | 0.313 | 0.318 | 0.434 | 0.348 | 0.705 |
| Cristóbal Colón | 0.708 | 0.827 | 0.809 | 0.786 | 0.582 | 0.814 | 2.156 | 0.233 | 0.269 | 0.344 | 0.374 | 0.303 | 0.651 |
| Simón Bolívar | 0.691 | 0.813 | 0.782 | 0.772 | 0.586 | 0.783 | 0.254 | 3 | **-0.011* | **0.095* | **0.057* | **0.035* | 0.298 |
| Puerto Quito | 0.691 | 0.828 | 0.809 | 0.779 | 0.569 | 0.804 | 0.293 | **0.016* | 1.857 | **0.209* | **0.238* | **0.064* | 0.416 |
| Cube | 0.694 | 0.847 | 0.849 | 0.811 | 0.583 | 0.831 | 0.273 | **0.028* | **0.058* | 0.762 | **0.129* | **0.010* | 0.348 |
| Quingüe | 0.714 | 0.858 | 0.868 | 0.831 | 0.588 | 0.847 | 0.386 | **0.077* | **0.114* | **0.046* | 0.429 | **0.025* | **0.227* |
| Santo Domingo | 0.71 | 0.853 | 0.859 | 0.823 | 0.587 | 0.84 | 0.357 | **0.102* | **0.219* | **0.105* | **0.114* | 0.679 | 0.28 |
| La Maná | 0.766 | 0.89 | 0.916 | 0.885 | 0.605 | 0.891 | 0.479 | **0.152* | 0.35 | 0.226 | **0.227* | 0.175 | 0 |

**Supplementary Table 4**

Mantel tests for each mitochondrial gene. Mantel R: Mantel coefficient; p-val1: one-tailed p-value (H_o_: R ≤ 0); p-val2: one-tailed p-value (H_o_: R ≥ 0); p-val3: two-tailed p-value (H_o_: R = 0); llim 2.5%: lower confidence limit; ulim: upper confidence limit. P-values were calculated after 20000 permutations.

| **Gene** | **Mantel R** | **p-val1** | **p-val2** | **p-val3** | **llim 2.5%** | **ulim 97.5%** |
| --- | --- | --- | --- | --- | --- | --- |
| CO1 | 0.684 | 5.10^-5^ | 1 | 5.10^-5^ | 0.606 | 0.713 |
| 16S | 0.635 | 5.10^-5^ | 1 | 5.10^-5^ | 0.536 | 0.666 |
| 12S | 0.584 | 5.10^-5^ | 1 | 5.10^-5^ | 0.407 | 0.628 |

**Supplementary Table 5**

Differentiation between population of *Oophaga sylvatica* by pairwise F_ST_ (upper diagonal) for the ddRAD data set composed of 3,785 SNPs.

| F_ST_ | Durango | Lita | Alto Tambo | Otokiki | Felfa | Cristóbal  Colón | Simón  Bolívar | Puerto Quito | Cube | Quingüe | Santo Domingo | La Maná |
| --- | --- | --- | --- | --- | --- | --- | --- | --- | --- | --- | --- | --- |
| San Antonio | 0.0163 | 0.0141 | 0.0151 | 0.0162 | 0.0303 | 0.0367 | 0.0480 | 0.0654 | 0.0449 | 0.0597 | 0.0671 | 0.0607 |
| Durango |  | 0.0101 | 0.0059 | 0.0063 | 0.0258 | 0.0353 | 0.0430 | 0.0552 | 0.0450 | 0.0579 | 0.0611 | 0.0546 |
| Lita |  |  | 0.0140 | 0.0072 | 0.0424 | 0.0569 | 0.0664 | 0.0840 | 0.0678 | 0.0937 | 0.0974 | 0.0876 |
| Alto  Tambo |  |  |  | 0.0082 | 0.0401 | 0.0512 | 0.0590 | 0.0826 | 0.0585 | 0.0950 | 0.0966 | 0.0823 |
| Otokiki |  |  |  |  | 0.0247 | 0.0314 | 0.0338 | 0.0330 | 0.0322 | 0.0300 | 0.0349 | 0.0327 |
| Felfa |  |  |  |  |  | 0.0295 | 0.0197 | 0.0466 | 0.0388 | 0.0724 | 0.0680 | 0.0498 |
| Cristóbal  Colón | |  |  |  |  |  | 0.0262 | 0.0636 | 0.0512 | 0.0832 | 0.0893 | 0.0658 |
| Simón  Bolívar | |  |  |  |  |  |  | 0.0552 | 0.0502 | 0.1030 | 0.0847 | 0.0597 |
| Puerto Quito |  |  |  |  |  |  |  |  | 0.0678 | 0.1370 | 0.0873 | 0.0894 |
| Cube |  |  |  |  |  |  |  |  |  | 0.0702 | 0.0537 | 0.0425 |
| Quingüe |  |  |  |  |  |  |  |  |  |  | 0.1222 | 0.0965 |
| Santo  Domingo | |  |  |  |  |  |  |  |  |  |  | 0.0546 |
